# Electron reconstruction and identification efficiency measurements with the ATLAS detector using the 2011 LHC proton–proton collision data

**DOI:** 10.1140/epjc/s10052-014-2941-0

**Published:** 2014-07-15

**Authors:** The ATLAS Collaboration, G. Aad, T. Abajyan, B. Abbott, J. Abdallah, S. Abdel Khalek, O. Abdinov, R. Aben, B. Abi, M. Abolins, O. S. AbouZeid, H. Abramowicz, H. Abreu, Y. Abulaiti, B. S. Acharya, L. Adamczyk, D. L. Adams, T. N. Addy, J. Adelman, S. Adomeit, T. Adye, T. Agatonovic-Jovin, J. A. Aguilar-Saavedra, M. Agustoni, S. P. Ahlen, A. Ahmad, F. Ahmadov, G. Aielli, T. P. A. Åkesson, G. Akimoto, A. V. Akimov, J. Albert, S. Albrand, M. J. Alconada Verzini, M. Aleksa, I. N. Aleksandrov, C. Alexa, G. Alexander, G. Alexandre, T. Alexopoulos, M. Alhroob, G. Alimonti, L. Alio, J. Alison, B. M. M. Allbrooke, L. J. Allison, P. P. Allport, S. E. Allwood-Spiers, J. Almond, A. Aloisio, R. Alon, A. Alonso, F. Alonso, C. Alpigiani, A. Altheimer, B. Alvarez Gonzalez, M. G. Alviggi, K. Amako, Y. Amaral Coutinho, C. Amelung, D. Amidei, V. V. Ammosov, S. P. Amor Dos Santos, A. Amorim, S. Amoroso, N. Amram, G. Amundsen, C. Anastopoulos, L. S. Ancu, N. Andari, T. Andeen, C. F. Anders, G. Anders, K. J. Anderson, A. Andreazza, V. Andrei, X. S. Anduaga, S. Angelidakis, P. Anger, A. Angerami, F. Anghinolfi, A. V. Anisenkov, N. Anjos, A. Annovi, A. Antonaki, M. Antonelli, A. Antonov, J. Antos, F. Anulli, M. Aoki, L. Aperio Bella, R. Apolle, G. Arabidze, I. Aracena, Y. Arai, J. P. Araque, A. T. H. Arce, J-F. Arguin, S. Argyropoulos, M. Arik, A. J. Armbruster, O. Arnaez, V. Arnal, O. Arslan, A. Artamonov, G. Artoni, S. Asai, N. Asbah, A. Ashkenazi, S. Ask, B. Åsman, L. Asquith, K. Assamagan, R. Astalos, M. Atkinson, N. B. Atlay, B. Auerbach, E. Auge, K. Augsten, M. Aurousseau, G. Avolio, G. Azuelos, Y. Azuma, M. A. Baak, C. Bacci, A. M. Bach, H. Bachacou, K. Bachas, M. Backes, M. Backhaus, J. Backus Mayes, E. Badescu, P. Bagiacchi, P. Bagnaia, Y. Bai, D. C. Bailey, T. Bain, J. T. Baines, O. K. Baker, S. Baker, P. Balek, F. Balli, E. Banas, Sw. Banerjee, D. Banfi, A. Bangert, A. A. E. Bannoura, V. Bansal, H. S. Bansil, L. Barak, S. P. Baranov, T. Barber, E. L. Barberio, D. Barberis, M. Barbero, T. Barillari, M. Barisonzi, T. Barklow, N. Barlow, B. M. Barnett, R. M. Barnett, Z. Barnovska, A. Baroncelli, G. Barone, A. J. Barr, F. Barreiro, J. Barreiro Guimarães da Costa, R. Bartoldus, A. E. Barton, P. Bartos, V. Bartsch, A. Bassalat, A. Basye, R. L. Bates, L. Batkova, J. R. Batley, M. Battistin, F. Bauer, H. S. Bawa, T. Beau, P. H. Beauchemin, R. Beccherle, P. Bechtle, H. P. Beck, K. Becker, S. Becker, M. Beckingham, C. Becot, A. J. Beddall, A. Beddall, S. Bedikian, V. A. Bednyakov, C. P. Bee, L. J. Beemster, T. A. Beermann, M. Begel, K. Behr, C. Belanger-Champagne, P. J. Bell, W. H. Bell, G. Bella, L. Bellagamba, A. Bellerive, M. Bellomo, A. Belloni, K. Belotskiy, O. Beltramello, O. Benary, D. Benchekroun, K. Bendtz, N. Benekos, Y. Benhammou, E. Benhar Noccioli, J. A. Benitez Garcia, D. P. Benjamin, J. R. Bensinger, K. Benslama, S. Bentvelsen, D. Berge, E. Bergeaas Kuutmann, N. Berger, F. Berghaus, E. Berglund, J. Beringer, C. Bernard, P. Bernat, C. Bernius, F. U. Bernlochner, T. Berry, P. Berta, C. Bertella, F. Bertolucci, M. I. Besana, G. J. Besjes, O. Bessidskaia, N. Besson, C. Betancourt, S. Bethke, W. Bhimji, R. M. Bianchi, L. Bianchini, M. Bianco, O. Biebel, S. P. Bieniek, K. Bierwagen, J. Biesiada, M. Biglietti, J. Bilbao De Mendizabal, H. Bilokon, M. Bindi, S. Binet, A. Bingul, C. Bini, C. W. Black, J. E. Black, K. M. Black, D. Blackburn, R. E. Blair, J.-B. Blanchard, T. Blazek, I. Bloch, C. Blocker, W. Blum, U. Blumenschein, G. J. Bobbink, V. S. Bobrovnikov, S. S. Bocchetta, A. Bocci, C. R. Boddy, M. Boehler, J. Boek, T. T. Boek, J. A. Bogaerts, A. G. Bogdanchikov, A. Bogouch, C. Bohm, J. Bohm, V. Boisvert, T. Bold, V. Boldea, A. S. Boldyrev, N. M. Bolnet, M. Bomben, M. Bona, M. Boonekamp, A. Borisov, G. Borissov, M. Borri, S. Borroni, J. Bortfeldt, V. Bortolotto, K. Bos, D. Boscherini, M. Bosman, H. Boterenbrood, J. Boudreau, J. Bouffard, E. V. Bouhova-Thacker, D. Boumediene, C. Bourdarios, N. Bousson, S. Boutouil, A. Boveia, J. Boyd, I. R. Boyko, I. Bozovic-Jelisavcic, J. Bracinik, P. Branchini, A. Brandt, G. Brandt, O. Brandt, U. Bratzler, B. Brau, J. E. Brau, H. M. Braun, S. F. Brazzale, B. Brelier, K. Brendlinger, A. J. Brennan, R. Brenner, S. Bressler, K. Bristow, T. M. Bristow, D. Britton, F. M. Brochu, I. Brock, R. Brock, C. Bromberg, J. Bronner, G. Brooijmans, T. Brooks, W. K. Brooks, J. Brosamer, E. Brost, G. Brown, J. Brown, P. A. Bruckman de Renstrom, D. Bruncko, R. Bruneliere, S. Brunet, A. Bruni, G. Bruni, M. Bruschi, L. Bryngemark, T. Buanes, Q. Buat, F. Bucci, P. Buchholz, R. M. Buckingham, A. G. Buckley, S. I. Buda, I. A. Budagov, F. Buehrer, L. Bugge, M. K. Bugge, O. Bulekov, A. C. Bundock, H. Burckhart, S. Burdin, B. Burghgrave, S. Burke, I. Burmeister, E. Busato, V. Büscher, P. Bussey, C. P. Buszello, B. Butler, J. M. Butler, A. I. Butt, C. M. Buttar, J. M. Butterworth, P. Butti, W. Buttinger, A. Buzatu, M. Byszewski, S. Cabrera Urbán, D. Caforio, O. Cakir, P. Calafiura, G. Calderini, P. Calfayan, R. Calkins, L. P. Caloba, D. Calvet, S. Calvet, R. Camacho Toro, S. Camarda, D. Cameron, L. M. Caminada, R. Caminal Armadans, S. Campana, M. Campanelli, A. Campoverde, V. Canale, A. Canepa, J. Cantero, R. Cantrill, T. Cao, M. D. M. Capeans Garrido, I. Caprini, M. Caprini, M. Capua, R. Caputo, R. Cardarelli, T. Carli, G. Carlino, L. Carminati, S. Caron, E. Carquin, G. D. Carrillo-Montoya, A. A. Carter, J. R. Carter, J. Carvalho, D. Casadei, M. P. Casado, E. Castaneda-Miranda, A. Castelli, V. Castillo Gimenez, N. F. Castro, P. Catastini, A. Catinaccio, J. R. Catmore, A. Cattai, G. Cattani, S. Caughron, V. Cavaliere, D. Cavalli, M. Cavalli-Sforza, V. Cavasinni, F. Ceradini, B. Cerio, K. Cerny, A. S. Cerqueira, A. Cerri, L. Cerrito, F. Cerutti, M. Cerv, A. Cervelli, S. A. Cetin, A. Chafaq, D. Chakraborty, I. Chalupkova, K. Chan, P. Chang, B. Chapleau, J. D. Chapman, D. Charfeddine, D. G. Charlton, C. C. Chau, C. A. Chavez Barajas, S. Cheatham, A. Chegwidden, S. Chekanov, S. V. Chekulaev, G. A. Chelkov, M. A. Chelstowska, C. Chen, H. Chen, K. Chen, L. Chen, S. Chen, X. Chen, Y. Chen, H. C. Cheng, Y. Cheng, A. Cheplakov, R. Cherkaoui El Moursli, V. Chernyatin, E. Cheu, L. Chevalier, V. Chiarella, G. Chiefari, J. T. Childers, A. Chilingarov, G. Chiodini, A. S. Chisholm, R. T. Chislett, A. Chitan, M. V. Chizhov, S. Chouridou, B. K. B. Chow, I. A. Christidi, D. Chromek-Burckhart, M. L. Chu, J. Chudoba, L. Chytka, G. Ciapetti, A. K. Ciftci, R. Ciftci, D. Cinca, V. Cindro, A. Ciocio, P. Cirkovic, Z. H. Citron, M. Citterio, M. Ciubancan, A. Clark, P. J. Clark, R. N. Clarke, W. Cleland, J. C. Clemens, B. Clement, C. Clement, Y. Coadou, M. Cobal, A. Coccaro, J. Cochran, L. Coffey, J. G. Cogan, J. Coggeshall, B. Cole, S. Cole, A. P. Colijn, C. Collins-Tooth, J. Collot, T. Colombo, G. Colon, G. Compostella, P. Conde Muiño, E. Coniavitis, M. C. Conidi, S. H. Connell, I. A. Connelly, S. M. Consonni, V. Consorti, S. Constantinescu, C. Conta, G. Conti, F. Conventi, M. Cooke, B. D. Cooper, A. M. Cooper-Sarkar, N. J. Cooper-Smith, K. Copic, T. Cornelissen, M. Corradi, F. Corriveau, A. Corso-Radu, A. Cortes-Gonzalez, G. Cortiana, G. Costa, M. J. Costa, D. Costanzo, D. Côté, G. Cottin, G. Cowan, B. E. Cox, K. Cranmer, G. Cree, S. Crépé-Renaudin, F. Crescioli, M. Crispin Ortuzar, M. Cristinziani, G. Crosetti, C.-M. Cuciuc, C. Cuenca Almenar, T. Cuhadar Donszelmann, J. Cummings, M. Curatolo, C. Cuthbert, H. Czirr, P. Czodrowski, Z. Czyczula, S. D’Auria, M. D’Onofrio, M. J. Da Cunha Sargedas De Sousa, C. Da Via, W. Dabrowski, A. Dafinca, T. Dai, O. Dale, F. Dallaire, C. Dallapiccola, M. Dam, A. C. Daniells, M. Dano Hoffmann, V. Dao, G. Darbo, G. L. Darlea, S. Darmora, J. A. Dassoulas, W. Davey, C. David, T. Davidek, E. Davies, M. Davies, O. Davignon, A. R. Davison, P. Davison, Y. Davygora, E. Dawe, I. Dawson, R. K. Daya-Ishmukhametova, K. De, R. de Asmundis, S. De Castro, S. De Cecco, J. de Graat, N. De Groot, P. de Jong, C. De La Taille, H. De la Torre, F. De Lorenzi, L. De Nooij, D. De Pedis, A. De Salvo, U. De Sanctis, A. De Santo, J. B. De Vivie De Regie, G. De Zorzi, W. J. Dearnaley, R. Debbe, C. Debenedetti, B. Dechenaux, D. V. Dedovich, J. Degenhardt, I. Deigaard, J. Del Peso, T. Del Prete, F. Deliot, C. M. Delitzsch, M. Deliyergiyev, A. Dell’Acqua, L. Dell’Asta, M. Dell’Orso, M. Della Pietra, D. della Volpe, M. Delmastro, P. A. Delsart, C. Deluca, S. Demers, M. Demichev, A. Demilly, S. P. Denisov, D. Derendarz, J. E. Derkaoui, F. Derue, P. Dervan, K. Desch, C. Deterre, P. O. Deviveiros, A. Dewhurst, S. Dhaliwal, A. Di Ciaccio, L. Di Ciaccio, A. Di Domenico, C. Di Donato, A. Di Girolamo, B. Di Girolamo, A. Di Mattia, B. Di Micco, R. Di Nardo, A. Di Simone, R. Di Sipio, D. Di Valentino, M. A. Diaz, E.B. Diehl, J. Dietrich, T. A. Dietzsch, S. Diglio, A. Dimitrievska, J. Dingfelder, C. Dionisi, P. Dita, S. Dita, F. Dittus, F. Djama, T. Djobava, M. A. B. do Vale, A. Do Valle Wemans, T. K. O. Doan, D. Dobos, E. Dobson, C. Doglioni, T. Doherty, T. Dohmae, J. Dolejsi, Z. Dolezal, B. A. Dolgoshein, M. Donadelli, S. Donati, P. Dondero, J. Donini, J. Dopke, A. Doria, A. Dos Anjos, M. T. Dova, A. T. Doyle, M. Dris, J. Dubbert, S. Dube, E. Dubreuil, E. Duchovni, G. Duckeck, O. A. Ducu, D. Duda, A. Dudarev, F. Dudziak, L. Duflot, L. Duguid, M. Dührssen, M. Dunford, H. Duran Yildiz, M. Düren, A. Durglishvili, M. Dwuznik, M. Dyndal, J. Ebke, W. Edson, N. C. Edwards, W. Ehrenfeld, T. Eifert, G. Eigen, K. Einsweiler, T. Ekelof, M. El Kacimi, M. Ellert, S. Elles, F. Ellinghaus, N. Ellis, J. Elmsheuser, M. Elsing, D. Emeliyanov, Y. Enari, O. C. Endner, M. Endo, R. Engelmann, J. Erdmann, A. Ereditato, D. Eriksson, G. Ernis, J. Ernst, M. Ernst, J. Ernwein, D. Errede, S. Errede, E. Ertel, M. Escalier, H. Esch, C. Escobar, B. Esposito, A. I. Etienvre, E. Etzion, H. Evans, L. Fabbri, G. Facini, R. M. Fakhrutdinov, S. Falciano, J. Faltova, Y. Fang, M. Fanti, A. Farbin, A. Farilla, T. Farooque, S. Farrell, S. M. Farrington, P. Farthouat, F. Fassi, P. Fassnacht, D. Fassouliotis, A. Favareto, L. Fayard, P. Federic, O. L. Fedin, W. Fedorko, M. Fehling-Kaschek, S. Feigl, L. Feligioni, C. Feng, E. J. Feng, H. Feng, A. B. Fenyuk, S. Fernandez Perez, W. Fernando, S. Ferrag, J. Ferrando, V. Ferrara, A. Ferrari, P. Ferrari, R. Ferrari, D. E. Ferreira de Lima, A. Ferrer, D. Ferrere, C. Ferretti, A. Ferretto Parodi, M. Fiascaris, F. Fiedler, A. Filipčič, M. Filipuzzi, F. Filthaut, M. Fincke-Keeler, K. D. Finelli, M. C. N. Fiolhais, L. Fiorini, A. Firan, J. Fischer, M. J. Fisher, W. C. Fisher, E. A. Fitzgerald, M. Flechl, I. Fleck, P. Fleischmann, S. Fleischmann, G. T. Fletcher, G. Fletcher, T. Flick, A. Floderus, L. R. Flores Castillo, A. C. Florez Bustos, M. J. Flowerdew, A. Formica, A. Forti, D. Fortin, D. Fournier, H. Fox, S. Fracchia, P. Francavilla, M. Franchini, S. Franchino, D. Francis, M. Franklin, S. Franz, M. Fraternali, S. T. French, C. Friedrich, F. Friedrich, D. Froidevaux, J. A. Frost, C. Fukunaga, E. Fullana Torregrosa, B. G. Fulsom, J. Fuster, C. Gabaldon, O. Gabizon, A. Gabrielli, S. Gadatsch, S. Gadomski, G. Gagliardi, P. Gagnon, C. Galea, B. Galhardo, E. J. Gallas, V. Gallo, B. J. Gallop, P. Gallus, G. Galster, K. K. Gan, R. P. Gandrajula, J. Gao, Y. S. Gao, F. M. Garay Walls, F. Garberson, C. García, J. E. García Navarro, M. Garcia-Sciveres, R. W. Gardner, N. Garelli, V. Garonne, C. Gatti, G. Gaudio, B. Gaur, L. Gauthier, P. Gauzzi, I. L. Gavrilenko, C. Gay, G. Gaycken, E. N. Gazis, P. Ge, Z. Gecse, C. N. P. Gee, D. A. A. Geerts, Ch. Geich-Gimbel, K. Gellerstedt, C. Gemme, A. Gemmell, M. H. Genest, S. Gentile, M. George, S. George, D. Gerbaudo, A. Gershon, H. Ghazlane, N. Ghodbane, B. Giacobbe, S. Giagu, V. Giangiobbe, P. Giannetti, F. Gianotti, B. Gibbard, S. M. Gibson, M. Gilchriese, T. P. S. Gillam, D. Gillberg, G. Gilles, D. M. Gingrich, N. Giokaris, M. P. Giordani, R. Giordano, F. M. Giorgi, P. F. Giraud, D. Giugni, C. Giuliani, M. Giulini, B. K. Gjelsten, I. Gkialas, L. K. Gladilin, C. Glasman, J. Glatzer, P. C. F. Glaysher, A. Glazov, G. L. Glonti, M. Goblirsch-Kolb, J. R. Goddard, J. Godfrey, J. Godlewski, C. Goeringer, S. Goldfarb, T. Golling, D. Golubkov, A. Gomes, L. S. Gomez Fajardo, R. Gonçalo, J. Goncalves Pinto Firmino Da Costa, L. Gonella, S. González de la Hoz, G. Gonzalez Parra, M. L. Gonzalez Silva, S. Gonzalez-Sevilla, L. Goossens, P. A. Gorbounov, H. A. Gordon, I. Gorelov, G. Gorfine, B. Gorini, E. Gorini, A. Gorišek, E. Gornicki, A. T. Goshaw, C. Gössling, M. I. Gostkin, M. Gouighri, D. Goujdami, M. P. Goulette, A. G. Goussiou, C. Goy, S. Gozpinar, H. M. X. Grabas, L. Graber, I. Grabowska-Bold, P. Grafström, K-J. Grahn, J. Gramling, E. Gramstad, F. Grancagnolo, S. Grancagnolo, V. Grassi, V. Gratchev, H. M. Gray, E. Graziani, O. G. Grebenyuk, Z. D. Greenwood, K. Gregersen, I. M. Gregor, P. Grenier, J. Griffiths, N. Grigalashvili, A. A. Grillo, K. Grimm, S. Grinstein, Ph. Gris, Y. V. Grishkevich, J.-F. Grivaz, J. P. Grohs, A. Grohsjean, E. Gross, J. Grosse-Knetter, G. C. Grossi, J. Groth-Jensen, Z. J. Grout, K. Grybel, L. Guan, F. Guescini, D. Guest, O. Gueta, C. Guicheney, E. Guido, T. Guillemin, S. Guindon, U. Gul, C. Gumpert, J. Gunther, J. Guo, S. Gupta, P. Gutierrez, N. G. Gutierrez Ortiz, C. Gutschow, N. Guttman, C. Guyot, C. Gwenlan, C. B. Gwilliam, A. Haas, C. Haber, H. K. Hadavand, N. Haddad, P. Haefner, S. Hageboeck, Z. Hajduk, H. Hakobyan, M. Haleem, D. Hall, G. Halladjian, K. Hamacher, P. Hamal, K. Hamano, M. Hamer, A. Hamilton, S. Hamilton, P. G. Hamnett, L. Han, K. Hanagaki, K. Hanawa, M. Hance, P. Hanke, J. B. Hansen, J. D. Hansen, P. H. Hansen, K. Hara, A. S. Hard, T. Harenberg, S. Harkusha, D. Harper, R. D. Harrington, O. M. Harris, P. F. Harrison, F. Hartjes, A. Harvey, S. Hasegawa, Y. Hasegawa, A. Hasib, S. Hassani, S. Haug, M. Hauschild, R. Hauser, M. Havranek, C. M. Hawkes, R. J. Hawkings, A. D. Hawkins, T. Hayashi, D. Hayden, C. P. Hays, H. S. Hayward, S. J. Haywood, S. J. Head, T. Heck, V. Hedberg, L. Heelan, S. Heim, T. Heim, B. Heinemann, L. Heinrich, S. Heisterkamp, J. Hejbal, L. Helary, C. Heller, M. Heller, S. Hellman, D. Hellmich, C. Helsens, J. Henderson, R. C. W. Henderson, C. Hengler, A. Henrichs, A. M. Henriques Correia, S. Henrot-Versille, C. Hensel, G. H. Herbert, Y. Hernández Jiménez, R. Herrberg-Schubert, G. Herten, R. Hertenberger, L. Hervas, G. G. Hesketh, N. P. Hessey, R. Hickling, E. Higón-Rodriguez, J. C. Hill, K. H. Hiller, S. Hillert, S. J. Hillier, I. Hinchliffe, E. Hines, M. Hirose, D. Hirschbuehl, J. Hobbs, N. Hod, M. C. Hodgkinson, P. Hodgson, A. Hoecker, M. R. Hoeferkamp, J. Hoffman, D. Hoffmann, J. I. Hofmann, M. Hohlfeld, T. R. Holmes, T. M. Hong, L. Hooft van Huysduynen, J-Y. Hostachy, S. Hou, A. Hoummada, J. Howard, J. Howarth, M. Hrabovsky, I. Hristova, J. Hrivnac, T. Hryn’ova, P. J. Hsu, S.-C. Hsu, D. Hu, X. Hu, Y. Huang, Z. Hubacek, F. Hubaut, F. Huegging, T. B. Huffman, E. W. Hughes, G. Hughes, M. Huhtinen, T. A. Hülsing, M. Hurwitz, N. Huseynov, J. Huston, J. Huth, G. Iacobucci, G. Iakovidis, I. Ibragimov, L. Iconomidou-Fayard, J. Idarraga, E. Ideal, P. Iengo, O. Igonkina, T. Iizawa, Y. Ikegami, K. Ikematsu, M. Ikeno, D. Iliadis, N. Ilic, Y. Inamaru, T. Ince, P. Ioannou, M. Iodice, K. Iordanidou, V. Ippolito, A. Irles Quiles, C. Isaksson, M. Ishino, M. Ishitsuka, R. Ishmukhametov, C. Issever, S. Istin, J. M. Iturbe Ponce, A. V. Ivashin, W. Iwanski, H. Iwasaki, J. M. Izen, V. Izzo, B. Jackson, J. N. Jackson, M. Jackson, P. Jackson, M. R. Jaekel, V. Jain, K. Jakobs, S. Jakobsen, T. Jakoubek, J. Jakubek, D. O. Jamin, D. K. Jana, E. Jansen, H. Jansen, J. Janssen, M. Janus, G. Jarlskog, T. Javůrek, L. Jeanty, G.-Y. Jeng, D. Jennens, P. Jenni, J. Jentzsch, C. Jeske, S. Jézéquel, H. Ji, W. Ji, J. Jia, Y. Jiang, M. Jimenez Belenguer, S. Jin, A. Jinaru, O. Jinnouchi, M. D. Joergensen, K. E. Johansson, P. Johansson, K. A. Johns, K. Jon-And, G. Jones, R. W. L. Jones, T. J. Jones, J. Jongmanns, P. M. Jorge, K. D. Joshi, J. Jovicevic, X. Ju, C. A. Jung, R. M. Jungst, P. Jussel, A. Juste Rozas, M. Kaci, A. Kaczmarska, M. Kado, H. Kagan, M. Kagan, E. Kajomovitz, S. Kama, N. Kanaya, M. Kaneda, S. Kaneti, T. Kanno, V. A. Kantserov, J. Kanzaki, B. Kaplan, A. Kapliy, D. Kar, K. Karakostas, N. Karastathis, M. Karnevskiy, S. N. Karpov, K. Karthik, V. Kartvelishvili, A. N. Karyukhin, L. Kashif, G. Kasieczka, R. D. Kass, A. Kastanas, Y. Kataoka, A. Katre, J. Katzy, V. Kaushik, K. Kawagoe, T. Kawamoto, G. Kawamura, S. Kazama, V.F. Kazanin, M. Y. Kazarinov, R. Keeler, P. T. Keener, R. Kehoe, M. Keil, J. S. Keller, H. Keoshkerian, O. Kepka, B. P. Kerševan, S. Kersten, K. Kessoku, J. Keung, F. Khalil-zada, H. Khandanyan, A. Khanov, A. Khodinov, A. Khomich, T. J. Khoo, G. Khoriauli, A. Khoroshilov, V. Khovanskiy, E. Khramov, J. Khubua, H. Y. Kim, H. Kim, S. H. Kim, N. Kimura, O. Kind, B. T. King, M. King, R. S. B. King, S. B. King, J. Kirk, A. E. Kiryunin, T. Kishimoto, D. Kisielewska, F. Kiss, T. Kitamura, T. Kittelmann, K. Kiuchi, E. Kladiva, M. Klein, U. Klein, K. Kleinknecht, P. Klimek, A. Klimentov, R. Klingenberg, J. A. Klinger, E. B. Klinkby, T. Klioutchnikova, P. F. Klok, E.-E. Kluge, P. Kluit, S. Kluth, E. Kneringer, E. B. F. G. Knoops, A. Knue, T. Kobayashi, M. Kobel, M. Kocian, P. Kodys, P. Koevesarki, T. Koffas, E. Koffeman, L. A. Kogan, S. Kohlmann, Z. Kohout, T. Kohriki, T. Koi, H. Kolanoski, I. Koletsou, J. Koll, A. A. Komar, Y. Komori, T. Kondo, K. Köneke, A. C. König, S. König, T. Kono, R. Konoplich, N. Konstantinidis, R. Kopeliansky, S. Koperny, L. Köpke, A. K. Kopp, K. Korcyl, K. Kordas, A. Korn, A. A. Korol, I. Korolkov, E. V. Korolkova, V. A. Korotkov, O. Kortner, S. Kortner, V. V. Kostyukhin, S. Kotov, V. M. Kotov, A. Kotwal, C. Kourkoumelis, V. Kouskoura, A. Koutsman, R. Kowalewski, T. Z. Kowalski, W. Kozanecki, A. S. Kozhin, V. Kral, V. A. Kramarenko, G. Kramberger, D. Krasnopevtsev, M. W. Krasny, A. Krasznahorkay, J. K. Kraus, A. Kravchenko, S. Kreiss, M. Kretz, J. Kretzschmar, K. Kreutzfeldt, P. Krieger, K. Kroeninger, H. Kroha, J. Kroll, J. Kroseberg, J. Krstic, U. Kruchonak, H. Krüger, T. Kruker, N. Krumnack, Z. V. Krumshteyn, A. Kruse, M. C. Kruse, M. Kruskal, T. Kubota, S. Kuday, S. Kuehn, A. Kugel, A. Kuhl, T. Kuhl, V. Kukhtin, Y. Kulchitsky, S. Kuleshov, M. Kuna, J. Kunkle, A. Kupco, H. Kurashige, Y. A. Kurochkin, R. Kurumida, V. Kus, E. S. Kuwertz, M. Kuze, J. Kvita, A. La Rosa, L. La Rotonda, L. Labarga, C. Lacasta, F. Lacava, J. Lacey, H. Lacker, D. Lacour, V. R. Lacuesta, E. Ladygin, R. Lafaye, B. Laforge, T. Lagouri, S. Lai, H. Laier, L. Lambourne, S. Lammers, C. L. Lampen, W. Lampl, E. Lançon, U. Landgraf, M. P. J. Landon, V. S. Lang, C. Lange, A. J. Lankford, F. Lanni, K. Lantzsch, S. Laplace, C. Lapoire, J. F. Laporte, T. Lari, M. Lassnig, P. Laurelli, V. Lavorini, W. Lavrijsen, A. T. Law, P. Laycock, B. T. Le, O. Le Dortz, E. Le Guirriec, E. Le Menedeu, T. LeCompte, F. Ledroit-Guillon, C. A. Lee, H. Lee, J. S. H. Lee, S. C. Lee, L. Lee, G. Lefebvre, M. Lefebvre, F. Legger, C. Leggett, A. Lehan, M. Lehmacher, G. Lehmann Miotto, X. Lei, A. G. Leister, M. A. L. Leite, R. Leitner, D. Lellouch, B. Lemmer, K. J. C. Leney, T. Lenz, G. Lenzen, B. Lenzi, R. Leone, K. Leonhardt, S. Leontsinis, C. Leroy, C. G. Lester, C. M. Lester, J. Levêque, D. Levin, L. J. Levinson, M. Levy, A. Lewis, G. H. Lewis, A. M. Leyko, M. Leyton, B. Li, H. Li, H. L. Li, S. Li, X. Li, Y. Li, Z. Liang, H. Liao, B. Liberti, P. Lichard, K. Lie, J. Liebal, W. Liebig, C. Limbach, A. Limosani, M. Limper, S. C. Lin, F. Linde, B. E. Lindquist, J. T. Linnemann, E. Lipeles, A. Lipniacka, M. Lisovyi, T. M. Liss, D. Lissauer, A. Lister, A. M. Litke, B. Liu, D. Liu, J. B. Liu, K. Liu, L. Liu, M. Liu, Y. Liu, M. Livan, S. S. A. Livermore, A. Lleres, J. Llorente Merino, S. L. Lloyd, F. Lo Sterzo, E. Lobodzinska, P. Loch, W. S. Lockman, T. Loddenkoetter, F. K. Loebinger, A. E. Loevschall-Jensen, A. Loginov, C. W. Loh, T. Lohse, K. Lohwasser, M. Lokajicek, V. P. Lombardo, J. D. Long, R. E. Long, L. Lopes, D. Lopez Mateos, B. Lopez Paredes, J. Lorenz, N. Lorenzo Martinez, M. Losada, P. Loscutoff, M. J. Losty, X. Lou, A. Lounis, J. Love, P. A. Love, A. J. Lowe, F. Lu, H. J. Lubatti, C. Luci, A. Lucotte, F. Luehring, W. Lukas, L. Luminari, O. Lundberg, B. Lund-Jensen, M. Lungwitz, D. Lynn, R. Lysak, E. Lytken, H. Ma, L. L. Ma, G. Maccarrone, A. Macchiolo, B. Maček, J. Machado Miguens, D. Macina, D. Madaffari, R. Madar, H. J. Maddocks, W. F. Mader, A. Madsen, M. Maeno, T. Maeno, E. Magradze, K. Mahboubi, J. Mahlstedt, S. Mahmoud, C. Maiani, C. Maidantchik, A. Maio, S. Majewski, Y. Makida, N. Makovec, P. Mal, B. Malaescu, Pa. Malecki, V. P. Maleev, F. Malek, U. Mallik, D. Malon, C. Malone, S. Maltezos, V. M. Malyshev, S. Malyukov, J. Mamuzic, B. Mandelli, L. Mandelli, I. Mandić, R. Mandrysch, J. Maneira, A. Manfredini, L. Manhaes de Andrade Filho, J. A. Manjarres Ramos, A. Mann, P. M. Manning, A. Manousakis-Katsikakis, B. Mansoulie, R. Mantifel, L. Mapelli, L. March, J. F. Marchand, F. Marchese, G. Marchiori, M. Marcisovsky, C. P. Marino, C. N. Marques, F. Marroquim, S.P. Marsden, Z. Marshall, L. F. Marti, S. Marti-Garcia, B. Martin, J. P. Martin, T. A. Martin, V. J. Martin, B. Martin dit Latour, H. Martinez, M. Martinez, S. Martin-Haugh, A. C. Martyniuk, M. Marx, F. Marzano, A. Marzin, L. Masetti, T. Mashimo, R. Mashinistov, J. Masik, A. L. Maslennikov, I. Massa, N. Massol, P. Mastrandrea, A. Mastroberardino, T. Masubuchi, P. Matricon, H. Matsunaga, T. Matsushita, P. Mättig, S. Mättig, J. Mattmann, J. Maurer, S. J. Maxfield, D. A. Maximov, R. Mazini, L. Mazzaferro, G. Mc Goldrick, S. P. Mc Kee, A. McCarn, R. L. McCarthy, T. G. McCarthy, N. A. McCubbin, K. W. McFarlane, J. A. Mcfayden, G. Mchedlidze, T. Mclaughlan, S. J. McMahon, R. A. McPherson, A. Meade, J. Mechnich, M. Medinnis, S. Meehan, R. Meera-Lebbai, S. Mehlhase, A. Mehta, K. Meier, C. Meineck, B. Meirose, C. Melachrinos, B. R. Mellado Garcia, F. Meloni, L. Mendoza Navas, A. Mengarelli, S. Menke, E. Meoni, K. M. Mercurio, S. Mergelmeyer, N. Meric, P. Mermod, L. Merola, C. Meroni, F. S. Merritt, H. Merritt, A. Messina, J. Metcalfe, A. S. Mete, C. Meyer, C. Meyer, J-P. Meyer, J. Meyer, R. P. Middleton, S. Migas, L. Mijović, G. Mikenberg, M. Mikestikova, M. Mikuž, D. W. Miller, C. Mills, A. Milov, D. A. Milstead, D. Milstein, A. A. Minaenko, M. Miñano Moya, I. A. Minashvili, A. I. Mincer, B. Mindur, M. Mineev, Y. Ming, L. M. Mir, G. Mirabelli, T. Mitani, J. Mitrevski, V. A. Mitsou, S. Mitsui, A. Miucci, P. S. Miyagawa, J. U. Mjörnmark, T. Moa, K. Mochizuki, V. Moeller, S. Mohapatra, W. Mohr, S. Molander, R. Moles-Valls, K. Mönig, C. Monini, J. Monk, E. Monnier, J. Montejo Berlingen, F. Monticelli, S. Monzani, R. W. Moore, C. Mora Herrera, A. Moraes, N. Morange, J. Morel, D. Moreno, M. Moreno Llácer, P. Morettini, M. Morgenstern, M. Morii, S. Moritz, A. K. Morley, G. Mornacchi, J. D. Morris, L. Morvaj, H. G. Moser, M. Mosidze, J. Moss, R. Mount, E. Mountricha, S. V. Mouraviev, E. J. W. Moyse, S. Muanza, R. D. Mudd, F. Mueller, J. Mueller, K. Mueller, T. Mueller, D. Muenstermann, Y. Munwes, J. A. Murillo Quijada, W. J. Murray, E. Musto, A. G. Myagkov, M. Myska, O. Nackenhorst, J. Nadal, K. Nagai, R. Nagai, Y. Nagai, K. Nagano, A. Nagarkar, Y. Nagasaka, M. Nagel, A. M. Nairz, Y. Nakahama, K. Nakamura, T. Nakamura, I. Nakano, H. Namasivayam, G. Nanava, R. Narayan, T. Nattermann, T. Naumann, G. Navarro, R. Nayyar, H. A. Neal, P. Yu. Nechaeva, T. J. Neep, A. Negri, G. Negri, M. Negrini, S. Nektarijevic, A. Nelson, T. K. Nelson, S. Nemecek, P. Nemethy, A. A. Nepomuceno, M. Nessi, M. S. Neubauer, M. Neumann, R. M. Neves, P. Nevski, F. M. Newcomer, P. R. Newman, D. H. Nguyen, R. B. Nickerson, R. Nicolaidou, B. Nicquevert, J. Nielsen, N. Nikiforou, A. Nikiforov, V. Nikolaenko, I. Nikolic-Audit, K. Nikolics, K. Nikolopoulos, P. Nilsson, Y. Ninomiya, A. Nisati, R. Nisius, T. Nobe, L. Nodulman, M. Nomachi, I. Nomidis, S. Norberg, M. Nordberg, S. Nowak, M. Nozaki, L. Nozka, K. Ntekas, G. Nunes Hanninger, T. Nunnemann, E. Nurse, F. Nuti, B. J. O’Brien, F. O’grady, D. C. O’Neil, V. O’Shea, F. G. Oakham, H. Oberlack, T. Obermann, J. Ocariz, A. Ochi, M. I. Ochoa, S. Oda, S. Odaka, H. Ogren, A. Oh, S. H. Oh, C. C. Ohm, H. Ohman, T. Ohshima, W. Okamura, H. Okawa, Y. Okumura, T. Okuyama, A. Olariu, A. G. Olchevski, S. A. Olivares Pino, D. Oliveira Damazio, E. Oliver Garcia, D. Olivito, A. Olszewski, J. Olszowska, A. Onofre, P. U. E. Onyisi, C. J. Oram, M. J. Oreglia, Y. Oren, D. Orestano, N. Orlando, C. Oropeza Barrera, R. S. Orr, B. Osculati, R. Ospanov, G. Otero y Garzon, H. Otono, M. Ouchrif, E. A. Ouellette, F. Ould-Saada, A. Ouraou, K. P. Oussoren, Q. Ouyang, A. Ovcharova, M. Owen, V. E. Ozcan, N. Ozturk, K. Pachal, A. Pacheco Pages, C. Padilla Aranda, M. Pagáčová, S. Pagan Griso, E. Paganis, C. Pahl, F. Paige, P. Pais, K. Pajchel, G. Palacino, S. Palestini, D. Pallin, A. Palma, J. D. Palmer, Y. B. Pan, E. Panagiotopoulou, J. G. Panduro Vazquez, P. Pani, N. Panikashvili, S. Panitkin, D. Pantea, L. Paolozzi, Th. D. Papadopoulou, K. Papageorgiou, A. Paramonov, D. Paredes Hernandez, M. A. Parker, F. Parodi, J. A. Parsons, U. Parzefall, E. Pasqualucci, S. Passaggio, A. Passeri, F. Pastore, Fr. Pastore, G. Pásztor, S. Pataraia, N. D. Patel, J. R. Pater, S. Patricelli, T. Pauly, J. Pearce, M. Pedersen, S. Pedraza Lopez, R. Pedro, S. V. Peleganchuk, D. Pelikan, H. Peng, B. Penning, J. Penwell, D. V. Perepelitsa, E. Perez Codina, M. T. Pérez García-Estan, V. Perez Reale, L. Perini, H. Pernegger, R. Perrino, R. Peschke, V. D. Peshekhonov, K. Peters, R. F. Y. Peters, B. A. Petersen, J. Petersen, T. C. Petersen, E. Petit, A. Petridis, C. Petridou, E. Petrolo, F. Petrucci, M. Petteni, N. E. Pettersson, R. Pezoa, P. W. Phillips, G. Piacquadio, E. Pianori, A. Picazio, E. Piccaro, M. Piccinini, S. M. Piec, R. Piegaia, D. T. Pignotti, J. E. Pilcher, A. D. Pilkington, J. Pina, M. Pinamonti, A. Pinder, J. L. Pinfold, A. Pingel, B. Pinto, S. Pires, C. Pizio, M.-A. Pleier, V. Pleskot, E. Plotnikova, P. Plucinski, S. Poddar, F. Podlyski, R. Poettgen, L. Poggioli, D. Pohl, M. Pohl, G. Polesello, A. Policicchio, R. Polifka, A. Polini, C. S. Pollard, V. Polychronakos, K. Pommès, L. Pontecorvo, B. G. Pope, G. A. Popeneciu, D. S. Popovic, A. Poppleton, X. Portell Bueso, G. E. Pospelov, S. Pospisil, K. Potamianos, I. N. Potrap, C. J. Potter, C. T. Potter, G. Poulard, J. Poveda, V. Pozdnyakov, R. Prabhu, P. Pralavorio, A. Pranko, S. Prasad, R. Pravahan, S. Prell, D. Price, J. Price, L. E. Price, D. Prieur, M. Primavera, M. Proissl, K. Prokofiev, F. Prokoshin, E. Protopapadaki, S. Protopopescu, J. Proudfoot, M. Przybycien, H. Przysiezniak, E. Ptacek, E. Pueschel, D. Puldon, M. Purohit, P. Puzo, Y. Pylypchenko, J. Qian, G. Qin, A. Quadt, D. R. Quarrie, W. B. Quayle, D. Quilty, A. Qureshi, V. Radeka, V. Radescu, S. K. Radhakrishnan, P. Radloff, P. Rados, F. Ragusa, G. Rahal, S. Rajagopalan, M. Rammensee, M. Rammes, A. S. Randle-Conde, C. Rangel-Smith, K. Rao, F. Rauscher, T. C. Rave, T. Ravenscroft, M. Raymond, A. L. Read, D. M. Rebuzzi, A. Redelbach, G. Redlinger, R. Reece, K. Reeves, L. Rehnisch, A. Reinsch, H. Reisin, M. Relich, C. Rembser, Z. L. Ren, A. Renaud, M. Rescigno, S. Resconi, B. Resende, O. L. Rezanova, P. Reznicek, R. Rezvani, R. Richter, E. Richter-Was, M. Ridel, P. Rieck, M. Rijssenbeek, A. Rimoldi, L. Rinaldi, E. Ritsch, I. Riu, F. Rizatdinova, E. Rizvi, S. H. Robertson, A. Robichaud-Veronneau, D. Robinson, J. E. M. Robinson, A. Robson, C. Roda, L. Rodrigues, S. Roe, O. Røhne, S. Rolli, A. Romaniouk, M. Romano, G. Romeo, E. Romero Adam, N. Rompotis, L. Roos, E. Ros, S. Rosati, K. Rosbach, A. Rose, M. Rose, P. L. Rosendahl, O. Rosenthal, V. Rossetti, E. Rossi, L. P. Rossi, R. Rosten, M. Rotaru, I. Roth, J. Rothberg, D. Rousseau, C. R. Royon, A. Rozanov, Y. Rozen, X. Ruan, F. Rubbo, I. Rubinskiy, V. I. Rud, C. Rudolph, M. S. Rudolph, F. Rühr, A. Ruiz-Martinez, Z. Rurikova, N. A. Rusakovich, A. Ruschke, J. P. Rutherfoord, N. Ruthmann, Y. F. Ryabov, M. Rybar, G. Rybkin, N. C. Ryder, A. F. Saavedra, S. Sacerdoti, A. Saddique, I. Sadeh, H. F-W. Sadrozinski, R. Sadykov, F. Safai Tehrani, H. Sakamoto, Y. Sakurai, G. Salamanna, A. Salamon, M. Saleem, D. Salek, P. H. Sales De Bruin, D. Salihagic, A. Salnikov, J. Salt, B. M. Salvachua Ferrando, D. Salvatore, F. Salvatore, A. Salvucci, A. Salzburger, D. Sampsonidis, A. Sanchez, J. Sánchez, V. Sanchez Martinez, H. Sandaker, H. G. Sander, M. P. Sanders, M. Sandhoff, T. Sandoval, C. Sandoval, R. Sandstroem, D. P. C. Sankey, A. Sansoni, C. Santoni, R. Santonico, H. Santos, I. Santoyo Castillo, K. Sapp, A. Sapronov, J. G. Saraiva, B. Sarrazin, G. Sartisohn, O. Sasaki, Y. Sasaki, I. Satsounkevitch, G. Sauvage, E. Sauvan, P. Savard, D. O. Savu, C. Sawyer, L. Sawyer, J. Saxon, C. Sbarra, A. Sbrizzi, T. Scanlon, D. A. Scannicchio, M. Scarcella, J. Schaarschmidt, P. Schacht, D. Schaefer, R. Schaefer, A. Schaelicke, S. Schaepe, S. Schaetzel, U. Schäfer, A. C. Schaffer, D. Schaile, R. D. Schamberger, V. Scharf, V. A. Schegelsky, D. Scheirich, M. Schernau, M. I. Scherzer, C. Schiavi, J. Schieck, C. Schillo, M. Schioppa, S. Schlenker, E. Schmidt, K. Schmieden, C. Schmitt, S. Schmitt, B. Schneider, Y. J. Schnellbach, U. Schnoor, L. Schoeffel, A. Schoening, B. D. Schoenrock, A. L. S. Schorlemmer, M. Schott, D. Schouten, J. Schovancova, M. Schram, S. Schramm, M. Schreyer, C. Schroeder, N. Schuh, M. J. Schultens, H.-C. Schultz-Coulon, H. Schulz, M. Schumacher, B. A. Schumm, Ph. Schune, A. Schwartzman, Ph. Schwegler, Ph. Schwemling, R. Schwienhorst, J. Schwindling, T. Schwindt, M. Schwoerer, F. G. Sciacca, E. Scifo, G. Sciolla, W. G. Scott, F. Scuri, F. Scutti, J. Searcy, G. Sedov, E. Sedykh, S. C. Seidel, A. Seiden, F. Seifert, J. M. Seixas, G. Sekhniaidze, S. J. Sekula, K. E. Selbach, D. M. Seliverstov, G. Sellers, N. Semprini-Cesari, C. Serfon, L. Serin, L. Serkin, T. Serre, R. Seuster, H. Severini, F. Sforza, A. Sfyrla, E. Shabalina, M. Shamim, L. Y. Shan, J. T. Shank, Q. T. Shao, M. Shapiro, P. B. Shatalov, K. Shaw, P. Sherwood, S. Shimizu, C. O. Shimmin, M. Shimojima, M. Shiyakova, A. Shmeleva, M. J. Shochet, D. Short, S. Shrestha, E. Shulga, M. A. Shupe, S. Shushkevich, P. Sicho, D. Sidorov, A. Sidoti, F. Siegert, Dj. Sijacki, O. Silbert, J. Silva, Y. Silver, D. Silverstein, S. B. Silverstein, V. Simak, O. Simard, Lj. Simic, S. Simion, E. Simioni, B. Simmons, R. Simoniello, M. Simonyan, P. Sinervo, N. B. Sinev, V. Sipica, G. Siragusa, A. Sircar, A. N. Sisakyan, S. Yu. Sivoklokov, J. Sjölin, T. B. Sjursen, L. A. Skinnari, H. P. Skottowe, K. Yu. Skovpen, P. Skubic, M. Slater, T. Slavicek, K. Sliwa, V. Smakhtin, B. H. Smart, L. Smestad, S. Yu. Smirnov, Y. Smirnov, L. N. Smirnova, O. Smirnova, M. Smizanska, K. Smolek, A. A. Snesarev, G. Snidero, J. Snow, S. Snyder, R. Sobie, F. Socher, J. Sodomka, A. Soffer, D. A. Soh, C. A. Solans, M. Solar, J. Solc, E. Yu. Soldatov, U. Soldevila, E. Solfaroli Camillocci, A. A. Solodkov, O. V. Solovyanov, V. Solovyev, P. Sommer, H. Y. Song, N. Soni, A. Sood, V. Sopko, B. Sopko, V. Sorin, M. Sosebee, R. Soualah, P. Soueid, A. M. Soukharev, D. South, S. Spagnolo, F. Spanò, W. R. Spearman, R. Spighi, G. Spigo, M. Spousta, T. Spreitzer, B. Spurlock, R. D. St. Denis, S. Staerz, J. Stahlman, R. Stamen, E. Stanecka, R. W. Stanek, C. Stanescu, M. Stanescu-Bellu, M. M. Stanitzki, S. Stapnes, E. A. Starchenko, J. Stark, P. Staroba, P. Starovoitov, R. Staszewski, P. Stavina, G. Steele, P. Steinberg, I. Stekl, B. Stelzer, H. J. Stelzer, O. Stelzer-Chilton, H. Stenzel, S. Stern, G. A. Stewart, J. A. Stillings, M. C. Stockton, M. Stoebe, K. Stoerig, G. Stoicea, P. Stolte, S. Stonjek, A. R. Stradling, A. Straessner, J. Strandberg, S. Strandberg, A. Strandlie, E. Strauss, M. Strauss, P. Strizenec, R. Ströhmer, D. M. Strom, R. Stroynowski, S. A. Stucci, B. Stugu, N. A. Styles, D. Su, J. Su, HS. Subramania, R. Subramaniam, A. Succurro, Y. Sugaya, C. Suhr, M. Suk, V. V. Sulin, S. Sultansoy, T. Sumida, X. Sun, J. E. Sundermann, K. Suruliz, G. Susinno, M. R. Sutton, Y. Suzuki, M. Svatos, S. Swedish, M. Swiatlowski, I. Sykora, T. Sykora, D. Ta, K. Tackmann, J. Taenzer, A. Taffard, R. Tafirout, N. Taiblum, Y. Takahashi, H. Takai, R. Takashima, H. Takeda, T. Takeshita, Y. Takubo, M. Talby, A. A. Talyshev, J. Y. C. Tam, M. C. Tamsett, K. G. Tan, J. Tanaka, R. Tanaka, S. Tanaka, S. Tanaka, A. J. Tanasijczuk, K. Tani, N. Tannoury, S. Tapprogge, S. Tarem, F. Tarrade, G. F. Tartarelli, P. Tas, M. Tasevsky, T. Tashiro, E. Tassi, A. Tavares Delgado, Y. Tayalati, C. Taylor, F. E. Taylor, G. N. Taylor, W. Taylor, F. A. Teischinger, M. Teixeira Dias Castanheira, P. Teixeira-Dias, K. K. Temming, H. Ten Kate, P. K. Teng, S. Terada, K. Terashi, J. Terron, S. Terzo, M. Testa, R. J. Teuscher, J. Therhaag, T. Theveneaux-Pelzer, S. Thoma, J. P. Thomas, J. Thomas-Wilsker, E. N. Thompson, P. D. Thompson, P. D. Thompson, A. S. Thompson, L. A. Thomsen, E. Thomson, M. Thomson, W. M. Thong, R. P. Thun, F. Tian, M. J. Tibbetts, V. O. Tikhomirov, Yu. A. Tikhonov, S. Timoshenko, E. Tiouchichine, P. Tipton, S. Tisserant, T. Todorov, S. Todorova-Nova, B. Toggerson, J. Tojo, S. Tokár, K. Tokushuku, K. Tollefson, L. Tomlinson, M. Tomoto, L. Tompkins, K. Toms, N. D. Topilin, E. Torrence, H. Torres, E. Torró Pastor, J. Toth, F. Touchard, D. R. Tovey, H. L. Tran, T. Trefzger, L. Tremblet, A. Tricoli, I. M. Trigger, S. Trincaz-Duvoid, M. F. Tripiana, N. Triplett, W. Trischuk, B. Trocmé, C. Troncon, M. Trottier-McDonald, M. Trovatelli, P. True, M. Trzebinski, A. Trzupek, C. Tsarouchas, J. C-L. Tseng, P. V. Tsiareshka, D. Tsionou, G. Tsipolitis, N. Tsirintanis, S. Tsiskaridze, V. Tsiskaridze, E. G. Tskhadadze, I. I. Tsukerman, V. Tsulaia, S. Tsuno, D. Tsybychev, A. Tua, A. Tudorache, V. Tudorache, A. N. Tuna, S. A. Tupputi, S. Turchikhin, D. Turecek, I. Turk Cakir, R. Turra, P. M. Tuts, A. Tykhonov, M. Tylmad, M. Tyndel, K. Uchida, I. Ueda, R. Ueno, M. Ughetto, M. Ugland, M. Uhlenbrock, F. Ukegawa, G. Unal, A. Undrus, G. Unel, F. C. Ungaro, Y. Unno, D. Urbaniec, P. Urquijo, G. Usai, A. Usanova, L. Vacavant, V. Vacek, B. Vachon, N. Valencic, S. Valentinetti, A. Valero, L. Valery, S. Valkar, E. Valladolid Gallego, S. Vallecorsa, J. A. Valls Ferrer, R. Van Berg, P. C. Van Der Deijl, R. van der Geer, H. van der Graaf, R. Van Der Leeuw, D. van der Ster, N. van Eldik, P. van Gemmeren, J. Van Nieuwkoop, I. van Vulpen, M. C. van Woerden, M. Vanadia, W. Vandelli, A. Vaniachine, P. Vankov, F. Vannucci, G. Vardanyan, R. Vari, E. W. Varnes, T. Varol, D. Varouchas, A. Vartapetian, K. E. Varvell, F. Vazeille, T. Vazquez Schroeder, J. Veatch, F. Veloso, S. Veneziano, A. Ventura, D. Ventura, M. Venturi, N. Venturi, A. Venturini, V. Vercesi, M. Verducci, W. Verkerke, J. C. Vermeulen, A. Vest, M. C. Vetterli, O. Viazlo, I. Vichou, T. Vickey, O. E. Vickey Boeriu, G. H. A. Viehhauser, S. Viel, R. Vigne, M. Villa, M. Villaplana Perez, E. Vilucchi, M. G. Vincter, V. B. Vinogradov, J. Virzi, O. Vitells, I. Vivarelli, F. Vives Vaque, S. Vlachos, D. Vladoiu, M. Vlasak, A. Vogel, P. Vokac, G. Volpi, M. Volpi, H. von der Schmitt, H. von Radziewski, E. von Toerne, V. Vorobel, K. Vorobev, M. Vos, R. Voss, J.H. Vossebeld, N. Vranjes, M. Vranjes Milosavljevic, V. Vrba, M. Vreeswijk, T. Vu Anh, R. Vuillermet, I. Vukotic, Z. Vykydal, W. Wagner, P. Wagner, S. Wahrmund, J. Wakabayashi, J. Walder, R. Walker, W. Walkowiak, R. Wall, P. Waller, B. Walsh, C. Wang, C. Wang, F. Wang, H. Wang, H. Wang, J. Wang, J. Wang, K. Wang, R. Wang, S. M. Wang, T. Wang, X. Wang, A. Warburton, C. P. Ward, D. R. Wardrope, M. Warsinsky, A. Washbrook, C. Wasicki, I. Watanabe, P. M. Watkins, A. T. Watson, I. J. Watson, M. F. Watson, G. Watts, S. Watts, B. M. Waugh, S. Webb, M. S. Weber, S. W. Weber, J. S. Webster, A. R. Weidberg, P. Weigell, B. Weinert, J. Weingarten, C. Weiser, H. Weits, P. S. Wells, T. Wenaus, D. Wendland, Z. Weng, T. Wengler, S. Wenig, N. Wermes, M. Werner, P. Werner, M. Wessels, J. Wetter, K. Whalen, A. White, M. J. White, R. White, S. White, D. Whiteson, D. Wicke, F. J. Wickens, W. Wiedenmann, M. Wielers, P. Wienemann, C. Wiglesworth, L. A. M. Wiik-Fuchs, P. A. Wijeratne, A. Wildauer, M. A. Wildt, H. G. Wilkens, J. Z. Will, H. H. Williams, S. Williams, C. Willis, S. Willocq, J. A. Wilson, A. Wilson, I. Wingerter-Seez, S. Winkelmann, F. Winklmeier, M. Wittgen, T. Wittig, J. Wittkowski, S. J. Wollstadt, M. W. Wolter, H. Wolters, B. K. Wosiek, J. Wotschack, M. J. Woudstra, K. W. Wozniak, M. Wright, M. Wu, S. L. Wu, X. Wu, Y. Wu, E. Wulf, T. R. Wyatt, B. M. Wynne, S. Xella, M. Xiao, D. Xu, L. Xu, B. Yabsley, S. Yacoob, M. Yamada, H. Yamaguchi, Y. Yamaguchi, A. Yamamoto, K. Yamamoto, S. Yamamoto, T. Yamamura, T. Yamanaka, K. Yamauchi, Y. Yamazaki, Z. Yan, H. Yang, H. Yang, U. K. Yang, Y. Yang, S. Yanush, L. Yao, W-M. Yao, Y. Yasu, E. Yatsenko, K. H. Yau Wong, J. Ye, S. Ye, A. L. Yen, E. Yildirim, M. Yilmaz, R. Yoosoofmiya, K. Yorita, R. Yoshida, K. Yoshihara, C. Young, C. J. S. Young, S. Youssef, D. R. Yu, J. Yu, J. M. Yu, J. Yu, L. Yuan, A. Yurkewicz, B. Zabinski, R. Zaidan, A. M. Zaitsev, A. Zaman, S. Zambito, L. Zanello, D. Zanzi, A. Zaytsev, C. Zeitnitz, M. Zeman, A. Zemla, K. Zengel, O. Zenin, T. Ženiš, D. Zerwas, G. Zevi della Porta, D. Zhang, F. Zhang, H. Zhang, J. Zhang, L. Zhang, X. Zhang, Z. Zhang, Z. Zhao, A. Zhemchugov, J. Zhong, B. Zhou, L. Zhou, N. Zhou, C. G. Zhu, H. Zhu, J. Zhu, Y. Zhu, X. Zhuang, A. Zibell, D. Zieminska, N. I. Zimine, C. Zimmermann, R. Zimmermann, S. Zimmermann, S. Zimmermann, Z. Zinonos, M. Ziolkowski, R. Zitoun, G. Zobernig, A. Zoccoli, M. zur Nedden, G. Zurzolo, V. Zutshi, L. Zwalinski

**Affiliations:** 1 Department of Physics, University of Adelaide, Adelaide, Australia; 2 Physics Department, SUNY Albany, Albany, NY USA; 3 Department of Physics, University of Alberta, Edmonton, AB Canada; 4 Department of Physics, Ankara University, Ankara, Turkey Department of Physics, Gazi University, Ankara, Turkey Division of Physics, TOBB University of Economics and Technology, Ankara, Turkey Turkish Atomic Energy Authority, Ankara, Turkey; 5 LAPP, CNRS/IN2P3 and Université de Savoie, Annecy-le-Vieux, France; 6 High Energy Physics Division, Argonne National Laboratory, Argonne, IL USA; 7 Department of Physics, University of Arizona, Tucson, AZ USA; 8 Department of Physics, The University of Texas at Arlington, Arlington, TX USA; 9 Physics Department, University of Athens, Athens, Greece; 10 Physics Department, National Technical University of Athens, Zografou, Athens, Greece; 11 Institute of Physics, Azerbaijan Academy of Sciences, Baku, Azerbaijan; 12 Institut de Física d’Altes Energies and Departament de Física de la Universitat Autònoma de Barcelona, Barcelona, Spain; 13 Institute of Physics, University of Belgrade, Belgrade, Serbia Vinca Institute of Nuclear Sciences, University of Belgrade, Belgrade, Serbia; 14 Department for Physics and Technology, University of Bergen, Bergen, Norway; 15 Physics Division, Lawrence Berkeley National Laboratory and University of California, Berkeley, CA USA; 16 Department of Physics, Humboldt University, Berlin, Germany; 17 Albert Einstein Center for Fundamental Physics and Laboratory for High Energy Physics, University of Bern, Bern, Switzerland; 18 School of Physics and Astronomy, University of Birmingham, Birmingham, UK; 19 Department of Physics, Bogazici University, Istanbul, Turkey Department of Physics, Dogus University, Istanbul, Turkey Department of Physics Engineering, Gaziantep University, Gaziantep, Turkey; 20 INFN Sezione di Bologna, Bologna, Italy Dipartimento di Fisica e Astronomia, Università di Bologna, Bologna, Italy; 21 Physikalisches Institut, University of Bonn, Bonn, Germany; 22 Department of Physics, Boston University, Boston, MA USA; 23 Department of Physics, Brandeis University, Waltham, MA USA; 24 Universidade Federal do Rio De Janeiro COPPE/EE/IF, Rio de Janeiro, Brazil Federal University of Juiz de Fora (UFJF), Juiz de Fora, Brazil Federal University of Sao Joao del Rei (UFSJ), Sao Joao del Rei, Brazil Instituto de Fisica, Universidade de Sao Paulo, São Paulo, Brazil; 25 Physics Department, Brookhaven National Laboratory, Upton, NY USA; 26 National Institute of Physics and Nuclear Engineering, Bucharest, Romania Physics Department, National Institute for Research and Development of Isotopic and Molecular Technologies, Cluj Napoca, Romania University Politehnica Bucharest, Bucharest, Romania West University in Timisoara, Timisoara, Romania; 27 Departamento de Física, Universidad de Buenos Aires, Buenos Aires, Argentina; 28 Cavendish Laboratory, University of Cambridge, Cambridge, UK; 29 Department of Physics, Carleton University, Ottawa, ON Canada; 30 CERN, Geneva, Switzerland; 31 Enrico Fermi Institute, University of Chicago, Chicago, IL USA; 32 Departamento de Física, Pontificia Universidad Católica de Chile, Santiago, Chile Departamento de Física, Universidad Técnica Federico Santa María, Valparaiso, Chile; 33 Institute of High Energy Physics, Chinese Academy of Sciences, Beijing, China Department of Modern Physics, University of Science and Technology of China, Hefei, Anhui, China Department of Physics, Nanjing University, Nanjing, Jiangsu, China School of Physics, Shandong University, Jinan, Shandong, China Physics Department, Shanghai Jiao Tong University, Shanghai, China; 34 Laboratoire de Physique Corpusculaire, Clermont Université and Université Blaise Pascal and CNRS/IN2P3, Clermont-Ferrand, France; 35 Nevis Laboratory, Columbia University, Irvington, NY USA; 36 Niels Bohr Institute, University of Copenhagen, Copenhagen, Denmark; 37 INFN Gruppo Collegato di Cosenza, Laboratori Nazionali di Frascati, Frascati, Italy Dipartimento di Fisica, Università della Calabria, Rende, Italy; 38 Faculty of Physics and Applied Computer Science, AGH University of Science and Technology, Kraków, Poland Marian Smoluchowski Institute of Physics, Jagiellonian University, Kraków, Poland; 39 The Henryk Niewodniczanski Institute of Nuclear Physics, Polish Academy of Sciences, Kraków, Poland; 40 Physics Department, Southern Methodist University, Dallas, TX USA; 41 Physics Department, University of Texas at Dallas, Richardson, TX USA; 42 DESY, Hamburg and Zeuthen, Germany; 43 Institut für Experimentelle Physik IV, Technische Universität Dortmund, Dortmund, Germany; 44 Institut für Kern- und Teilchenphysik, Technische Universität Dresden, Dresden, Germany; 45 Department of Physics, Duke University, Durham, NC USA; 46 SUPA-School of Physics and Astronomy, University of Edinburgh, Edinburgh, UK; 47 INFN Laboratori Nazionali di Frascati, Frascati, Italy; 48 Fakultät für Mathematik und Physik, Albert-Ludwigs-Universität, Freiburg, Germany; 49 Section de Physique, Université de Genève, Geneva, Switzerland; 50 INFN Sezione di Genova, Genoa, Italy Dipartimento di Fisica, Università di Genova, Genoa, Italy; 51 E. Andronikashvili Institute of Physics, Iv. Javakhishvili Tbilisi State University, Tbilisi, Georgia High Energy Physics Institute, Tbilisi State University, Tbilisi, Georgia; 52 II Physikalisches Institut, Justus-Liebig-Universität Giessen, Giessen, Germany; 53 SUPA-School of Physics and Astronomy, University of Glasgow, Glasgow, UK; 54 II Physikalisches Institut, Georg-August-Universität, Göttingen, Germany; 55 Laboratoire de Physique Subatomique et de Cosmologie, Université Grenoble-Alpes, CNRS/IN2P3, Grenoble, France; 56 Department of Physics, Hampton University, Hampton, VA USA; 57 Laboratory for Particle Physics and Cosmology, Harvard University, Cambridge, MA USA; 58 Kirchhoff-Institut für Physik, Ruprecht-Karls-Universität Heidelberg, Heidelberg, Germany Physikalisches Institut, Ruprecht-Karls-Universität Heidelberg, Heidelberg, Germany ZITI Institut für technische Informatik, Ruprecht-Karls-Universität Heidelberg, Mannheim, Germany; 59 Faculty of Applied Information Science, Hiroshima Institute of Technology, Hiroshima, Japan; 60 Department of Physics, Indiana University, Bloomington, IN USA; 61 Institut für Astro- und Teilchenphysik, Leopold-Franzens-Universität, Innsbruck, Austria; 62 University of Iowa, Iowa City, IA USA; 63 Department of Physics and Astronomy, Iowa State University, Ames, IA USA; 64 Joint Institute for Nuclear Research, JINR Dubna, Dubna, Russia; 65 KEK, High Energy Accelerator Research Organization, Tsukuba, Japan; 66 Graduate School of Science, Kobe University, Kobe, Japan; 67 Faculty of Science, Kyoto University, Kyoto, Japan; 68 Kyoto University of Education, Kyoto, Japan; 69 Department of Physics, Kyushu University, Fukuoka, Japan; 70 Instituto de Física La Plata, Universidad Nacional de La Plata and CONICET, La Plata, Argentina; 71 Physics Department, Lancaster University, Lancaster, UK; 72 INFN Sezione di Lecce, Lecce, Italy Dipartimento di Matematica e Fisica, Università del Salento, Lecce, Italy; 73 Oliver Lodge Laboratory, University of Liverpool, Liverpool, UK; 74 Department of Physics, Jožef Stefan Institute and University of Ljubljana, Ljubljana, Slovenia; 75 School of Physics and Astronomy, Queen Mary University of London, London, UK; 76 Department of Physics, Royal Holloway University of London, Surrey, UK; 77 Department of Physics and Astronomy, University College London, London, UK; 78 Louisiana Tech University, Ruston, LA USA; 79 Laboratoire de Physique Nucléaire et de Hautes Energies, UPMC and Université Paris-Diderot and CNRS/IN2P3, Paris, France; 80 Fysiska institutionen, Lunds universitet, Lund, Sweden; 81 Departamento de Fisica Teorica C-15, Universidad Autonoma de Madrid, Madrid, Spain; 82 Institut für Physik, Universität Mainz, Mainz, Germany; 83 School of Physics and Astronomy, University of Manchester, Manchester, UK; 84 CPPM, Aix-Marseille Université and CNRS/IN2P3, Marseille, France; 85 Department of Physics, University of Massachusetts, Amherst, MA USA; 86 Department of Physics, McGill University, Montreal, QC Canada; 87 School of Physics, University of Melbourne, Victoria, Australia; 88 Department of Physics, The University of Michigan, Ann Arbor, MI USA; 89 Department of Physics and Astronomy, Michigan State University, East Lansing, MI USA; 90 INFN Sezione di Milano, Milan, Italy Dipartimento di Fisica, Università di Milano, Milan, Italy; 91 B.I. Stepanov Institute of Physics, National Academy of Sciences of Belarus, Minsk, Republic of Belarus; 92 National Scientific and Educational Centre for Particle and High Energy Physics, Minsk, Republic of Belarus; 93 Department of Physics, Massachusetts Institute of Technology, Cambridge, MA USA; 94 Group of Particle Physics, University of Montreal, Montreal, QC Canada; 95 P.N. Lebedev Institute of Physics, Academy of Sciences, Moscow, Russia; 96 Institute for Theoretical and Experimental Physics (ITEP), Moscow, Russia; 97 Moscow Engineering and Physics Institute (MEPhI), Moscow, Russia; 98 D.V.Skobeltsyn Institute of Nuclear Physics, M.V.Lomonosov Moscow State University, Moscow, Russia; 99 Fakultät für Physik, Ludwig-Maximilians-Universität München, Munich, Germany; 100 Max-Planck-Institut für Physik (Werner-Heisenberg-Institut), Munich, Germany; 101 Nagasaki Institute of Applied Science, Nagasaki, Japan; 102 Graduate School of Science and Kobayashi-Maskawa Institute, Nagoya University, Nagoya, Japan; 103 INFN Sezione di Napoli, Naples, Italy Dipartimento di Fisica, Università di Napoli, Naples, Italy; 104 Department of Physics and Astronomy, University of New Mexico, Albuquerque, NM USA; 105 Institute for Mathematics, Astrophysics and Particle Physics, Radboud University Nijmegen/Nikhef, Nijmegen, The Netherlands; 106 Nikhef National Institute for Subatomic Physics and University of Amsterdam, Amsterdam, The Netherlands; 107 Department of Physics, Northern Illinois University, DeKalb, IL USA; 108 Budker Institute of Nuclear Physics, SB RAS, Novosibirsk, Russia; 109 Department of Physics, New York University, New York, NY USA; 110 Ohio State University, Columbus, OH USA; 111 Faculty of Science, Okayama University, Okayama, Japan; 112 Homer L. Dodge Department of Physics and Astronomy, University of Oklahoma, Norman, OK USA; 113 Department of Physics, Oklahoma State University, Stillwater, OK USA; 114 Palacký University, RCPTM, Olomouc, Czech Republic; 115 Center for High Energy Physics, University of Oregon, Eugene, OR USA; 116 LAL, Université Paris-Sud and CNRS/IN2P3, Orsay, France; 117 Graduate School of Science, Osaka University, Osaka, Japan; 118 Department of Physics, University of Oslo, Oslo, Norway; 119 Department of Physics, Oxford University, Oxford, UK; 120 INFN Sezione di Pavia, Pavia, Italy Dipartimento di Fisica, Università di Pavia, Pavia, Italy; 121 Department of Physics, University of Pennsylvania, Philadelphia, PA USA; 122 Petersburg Nuclear Physics Institute, Gatchina, Russia; 123 INFN Sezione di Pisa, Pisa, Italy Dipartimento di Fisica E. Fermi, Università di Pisa, Pisa, Italy; 124 Department of Physics and Astronomy, University of Pittsburgh, Pittsburgh, PA USA; 125 Laboratorio de Instrumentacao e Fisica Experimental de Particulas-LIP, Lisbon, Portugal Faculdade de Ciências, Universidade de Lisboa, Lisbon, Portugal Department of Physics, University of Coimbra, Coimbra, Portugal Centro de Física Nuclear da Universidade de Lisboa, Lisbon, Portugal Departamento de Fisica, Universidade do Minho, Braga, Portugal Departamento de Fisica Teorica y del Cosmos and CAFPE, Universidad de Granada, Granada, Spain Dep Fisica and CEFITEC of Faculdade de Ciencias e Tecnologia, Universidade Nova de Lisboa, Caparica, Portugal; 126 Institute of Physics, Academy of Sciences of the Czech Republic, Prague, Czech Republic; 127 Czech Technical University in Prague, Prague, Czech Republic; 128 Faculty of Mathematics and Physics, Charles University in Prague, Prague, Czech Republic; 129 State Research Center Institute for High Energy Physics, Protvino, Russia; 130 Particle Physics Department, Rutherford Appleton Laboratory, Didcot, UK; 131 Physics Department, University of Regina, Regina, SK Canada; 132 Ritsumeikan University, Kusatsu, Shiga Japan; 133 INFN Sezione di Roma, Rome, Italy Dipartimento di Fisica, Sapienza Università di Roma, Rome, Italy; 134 INFN Sezione di Roma Tor Vergata, Rome, Italy Dipartimento di Fisica, Università di Roma Tor Vergata, Rome, Italy; 135 INFN Sezione di Roma Tre, Rome, Italy Dipartimento di Matematica e Fisica, Università Roma Tre, Rome, Italy; 136 Faculté des Sciences Ain Chock, Réseau Universitaire de Physique des Hautes Energies-Université Hassan II, Casablanca, Morocco Centre National de l’Energie des Sciences Techniques Nucleaires, Rabat, Morocco Faculté des Sciences Semlalia, Université Cadi Ayyad, LPHEA-Marrakech, Marrakech, Morocco Faculté des Sciences, Université Mohamed Premier and LPTPM, Oujda, Morocco Faculté des sciences, Université Mohammed V-Agdal, Rabat, Morocco; 137 DSM/IRFU (Institut de Recherches sur les Lois Fondamentales de l’Univers), CEA Saclay (Commissariat à l’Energie Atomique et aux Energies Alternatives), Gif-sur-Yvette, France; 138 Santa Cruz Institute for Particle Physics, University of California Santa Cruz, Santa Cruz, CA USA; 139 Department of Physics, University of Washington, Seattle, WA USA; 140 Department of Physics and Astronomy, University of Sheffield, Sheffield, UK; 141 Department of Physics, Shinshu University, Nagano, Japan; 142 Fachbereich Physik, Universität Siegen, Siegen, Germany; 143 Department of Physics, Simon Fraser University, Burnaby, BC Canada; 144 SLAC National Accelerator Laboratory, Stanford, CA USA; 145 Faculty of Mathematics, Physics and Informatics, Comenius University, Bratislava, Slovak Republic Department of Subnuclear Physics, Institute of Experimental Physics of the Slovak Academy of Sciences, Kosice, Slovak Republic; 146 Department of Physics, University of Cape Town, Cape Town, South Africa Department of Physics, University of Johannesburg, Johannesburg, South Africa School of Physics, University of the Witwatersrand, Johannesburg, South Africa; 147 Department of Physics, Stockholm University, Stockholm, Sweden The Oskar Klein Centre, Stockholm, Sweden; 148 Physics Department, Royal Institute of Technology, Stockholm, Sweden; 149 Departments of Physics and Astronomy and Chemistry, Stony Brook University, Stony Brook, NY USA; 150 Department of Physics and Astronomy, University of Sussex, Brighton, UK; 151 School of Physics, University of Sydney, Sydney, Australia; 152 Institute of Physics, Academia Sinica, Taipei, Taiwan; 153 Department of Physics, Technion: Israel Institute of Technology, Haifa, Israel; 154 Raymond and Beverly Sackler School of Physics and Astronomy, Tel Aviv University, Tel Aviv, Israel; 155 Department of Physics, Aristotle University of Thessaloniki, Thessaloniki, Greece; 156 International Center for Elementary Particle Physics and Department of Physics, The University of Tokyo, Tokyo, Japan; 157 Graduate School of Science and Technology, Tokyo Metropolitan University, Tokyo, Japan; 158 Department of Physics, Tokyo Institute of Technology, Tokyo, Japan; 159 Department of Physics, University of Toronto, Toronto, ON Canada; 160 TRIUMF, Vancouver, BC, Canada Department of Physics and Astronomy, York University, Toronto, ON Canada; 161 Faculty of Pure and Applied Sciences, University of Tsukuba, Tsukuba, Japan; 162 Department of Physics and Astronomy, Tufts University, Medford, MA USA; 163 Centro de Investigaciones, Universidad Antonio Narino, Bogota, Colombia; 164 Department of Physics and Astronomy, University of California Irvine, Irvine, CA USA; 165 INFN Gruppo Collegato di Udine, Sezione di Trieste, Udine, Italy ICTP, Trieste, Italy Dipartimento di Chimica, Fisica e Ambiente, Università di Udine, Udine, Italy; 166 Department of Physics, University of Illinois, Urbana, IL USA; 167 Department of Physics and Astronomy, University of Uppsala, Uppsala, Sweden; 168 Instituto de Física Corpuscular (IFIC) and Departamento de Física Atómica Molecular y Nuclear and Departamento de Ingeniería Electrónica and Instituto de Microelectrónica de Barcelona (IMB-CNM), University of Valencia and CSIC, Valencia, Spain; 169 Department of Physics, University of British Columbia, Vancouver, BC Canada; 170 Department of Physics and Astronomy, University of Victoria, Victoria, BC Canada; 171 Department of Physics, University of Warwick, Coventry, UK; 172 Waseda University, Tokyo, Japan; 173 Department of Particle Physics, The Weizmann Institute of Science, Rehovot, Israel; 174 Department of Physics, University of Wisconsin, Madison, WI USA; 175 Fakultät für Physik und Astronomie, Julius-Maximilians-Universität, Würzburg, Germany; 176 Fachbereich C Physik, Bergische Universität Wuppertal, Wuppertal, Germany; 177 Department of Physics, Yale University, New Haven, CT USA; 178 Yerevan Physics Institute, Yerevan, Armenia; 179 Centre de Calcul de l’Institut National de Physique Nucléaire et de Physique des Particules (IN2P3), Villeurbanne, France; 180 CERN, 1211 Geneva 23, Switzerland

## Abstract

Many of the interesting physics processes to be measured at the LHC have a signature involving one or more isolated electrons. The electron reconstruction and identification efficiencies of the ATLAS detector at the LHC have been evaluated using proton–proton collision data collected in 2011 at $\sqrt{s}= 7$ TeV and corresponding to an integrated luminosity of 4.7 fb$^{-1}$. Tag-and-probe methods using events with leptonic decays of $W$ and $Z$ bosons and $J/\psi$ mesons are employed to benchmark these performance parameters. The combination of all measurements results in identification efficiencies determined with an accuracy at the few per mil level for electron transverse energy greater than 30 GeV.

## Introduction

The good performance of electron^1^ reconstruction and identification in the ATLAS experiment at the Large Hadron Collider (LHC) based at the CERN Laboratory has been an essential ingredient to its successful scientific programme. It has played a critical role in several analyses, as for instance in Standard Model measurements [1–4], the discovery of a Higgs boson [5], and the searches for new physics beyond the Standard Model [6]. Isolated electrons produced in many interesting physics processes can be subject to large backgrounds from misidentified hadrons, electrons from photon conversions, and non-isolated electrons originating from heavy-flavour decays. For this reason, it is important to efficiently reconstruct and identify electrons over the full acceptance of the detector, while at the same time to have a significant background rejection. In ATLAS, this is accomplished using a combination of powerful detector technologies: silicon detectors and a transition radiation tracker to identify the track of the electron and a longitudinally layered electromagnetic calorimeter system with fine lateral segmentation to measure the electron’s energy deposition, followed by hadronic calorimeters used to veto particles giving rise to significant hadronic activity.

During the 2011 data-taking period at $\sqrt{s}= 7$ TeV, the LHC steadily increased the instantaneous luminosity from $5 \times 10^{32}$ cm$^{-2}$ s$^{-1}$ to $3.7 \times 10^{33}$ cm$^{-2}$ s$^{-1}$, with an average superposition (“pile-up”) of approximately nine proton–proton interactions per beam crossing. In contrast to the electron performance goals for the 2010 period [7], which focused on robustness for the first LHC running, the goals for the 2011 period aimed at substantially increasing the background rejection power in this much busier environment to keep the online output rate of events triggered by electron signatures within its allocated budget while at the same time preserving high reconstruction and identification efficiencies for electrons. During this period, ATLAS collected large samples of isolated electrons from $W \rightarrow e\nu$, $Z \rightarrow ee$, and $J/\psi \rightarrow ee$ events, allowing precise measurements of the electron reconstruction and identification efficiencies over the range of transverse energies, $E_{\mathrm {T}}$, from 7 to 50 GeV. This paper reports on the methods used to perform these measurements, describes the improvements with respect to previous results [7], and benchmarks the performance of the 2011 electron reconstruction and identification used in various analyses performed with proton–proton collisions.

The structure of the paper is as follows. Section 2 provides a brief summary of the main components of the ATLAS detector. The electron trigger design, the algorithm for electron reconstruction and the electron identification criteria are described in Sect. 3. Section 4 focuses on the method used to compute the various efficiencies. The data and simulation samples used in this work are given in Sect. 5 together with the main triggers that enabled the event collection. Section 6 reports on the identification efficiency measurement, presenting the background evaluation and the results obtained with the tag-and-probe technique. A similar methodology, but using a subset of the samples available for the identification efficiency measurement, is used to extract the efficiency of the electron reconstruction described in Sect. 7. The study of the probability to mismeasure the charge of an electron is presented in Sect. 8. The summary of the work is given in Sect. 9.

## The ATLAS detector

The ATLAS detector is designed to observe particles produced in high-energy proton–proton and heavy-ion collisions. It is composed of an inner tracking detector (ID) immersed in a 2 T axial magnetic field produced by a thin superconducting solenoid, electromagnetic (EM) and hadronic calorimeters outside the solenoid, and air-core-toroid muon spectrometers. A three-level triggering system reduces the total data-taking rate from a bunch-crossing frequency of approximately 20 MHz to several hundred Hz. A detailed description of the detector is provided elsewhere [8]. In the following, only an overview of the main systems relevant to the results reported in this paper is provided.

The inner tracking detector provides precise reconstruction of tracks within a pseudorapidity range^2^ $|\eta | \lesssim 2.5$. The innermost part of the ID consists of a silicon pixel detector providing typically three measurement points for charged particles originating in the beam-interaction region. The closest layer to the beam-pipe (referred to as the b-layer) contributes significantly to precision vertexing and provides discrimination against photon conversions. A SemiConductor Tracker (SCT) consisting of modules with two layers of silicon micro-strip sensors surrounds the pixel detector, providing typically eight hits per track at intermediate radii. The outermost region of the ID is covered by a Transition Radiation Tracker (TRT) consisting of straw drift tubes filled with a Xenon mixture, interleaved with polypropylene/polyethylene transition radiators. For charged particles with transverse momentum $p_{\mathrm {T}}>0.5$ GeV within its pseudorapidity coverage ($|\eta | \lesssim 2$), the TRT provides typically 35 hits per track. The TRT offers additional electron identification capability via the detection of transition-radiation photons generated by the radiators.

The ATLAS calorimeter system has both electromagnetic and hadronic components and covers the pseudorapidity range $|\eta |<4.9$, with finer granularity over the region matched to the inner detector. The central EM calorimeters are of an accordion-geometry design made from lead/liquid-argon (LAr) detectors, providing a full $\phi$ coverage. These detectors are divided into two half-barrels ($-1.475<\eta <0$ and $0<\eta <1.475$) and two endcap (EMEC) components ($1.375<|\eta |<3.2$), with a transition region between the barrel and the endcaps ($1.37<|\eta |<1.52$) which contains a relatively large amount of inactive material. Over the region devoted to precision measurements ($|\eta |<2.47$, excluding the transition regions), the EM calorimeter is segmented into longitudinal (depth) compartments called *front* (also known as *strips*), *middle*, and *back*. The front layer consists of strips finely grained in the $\eta$ direction, offering excellent discrimination between photons and $\pi ^{0}\rightarrow \gamma \gamma$. At high electron or photon energy, most of the energy is collected in the middle layer, which has a lateral granularity of $0.025 \times 0.025$ in $(\eta ,\phi )$ space, while the back layer provides measurements of energy deposited in the tails of the shower. The hadronic calorimeters, which surround the EM detectors, provide additional discrimination through further energy measurements of possible shower tails. The central EM calorimeter is complemented by two presampler detectors in the region $|\eta |<1.52$ (barrel) and $1.5<|\eta |<1.8$ (endcaps), made of a thin LAr layer, providing a sampling for particles that start showering in front of the EM calorimeters. The forward calorimeter (FCal), a copper–tungsten/LAr detector, provides coverage at high pseudorapidity ($3.1<|\eta |<4.9$) with EM-shower identification capability given by its lateral granularity and longitudinal segmentation into three layers; this calorimeter plays an important role in extending the pseudorapidity range where electrons from $Z$-boson decays can be identified.

**Fig. 1:**
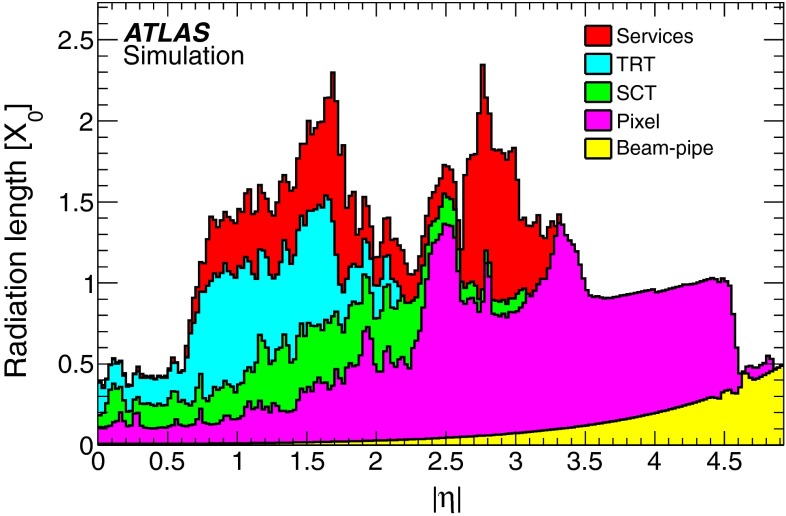
Amount of material in front of the cryostat, housing the solenoid and the EM calorimeters, in units of radiation length $X_{0}$, traversed by a particle as a function of $|\eta |$. The contributions of the different detector elements, including the services, are shown separately by *filled colour areas*

The inner detectors, including their services, as well as the cryostat containing the LAr calorimeter system correspond to a significant pseudorapidity-dependent amount of material located in front of the EM calorimeters and can impact the electron reconstruction and identification performance. Figure 1 shows the distribution of the material in front of the cryostat in terms of radiation lengths as a function of pseudorapidity. The observed material variations suggest a pseudorapidity-dependent optimisation of the selection criteria.

## Electron trigger, reconstruction, and identification

### Trigger

The trigger system in ATLAS [8, 9] comprises a hardware-based Level-1 trigger (L1) and software-based High-Level Triggers (HLT), composed of the Level-2 trigger (L2) and the Event Filter (EF). Inside the L1, the transverse energy $E_{\mathrm {T}}$ of electromagnetic showers collected in the calorimeters is computed within a granularity of $\varDelta \eta \times \varDelta \phi \approx 0.1 \times 0.1$. The selected objects must satisfy an $E_{\mathrm {T}}$ threshold and are used to seed the L2 reconstruction, which combines calorimetric and track information using fast algorithms. In the EF, offline-like algorithms are deployed for the reconstruction of the calorimetric quantities while an adapted version of the offline software is used to treat the information of the inner detector. During the 2011 run, the L1 output rate was kept below 60 kHz, the L2 rate below 5 kHz and the EF rate was approximately 400 Hz, averaged over the LHC fills.

### Reconstruction

#### Central electrons

The electron-reconstruction algorithm used in the central region of the detector equipped with the ID ($|\eta |<2.5$) identifies energy deposits in the EM calorimeter and associates these clusters of energy with reconstructed tracks in the inner detector. The three-step process is as follows.

*Cluster reconstruction:* EM clusters are seeded from energy deposits with total transverse energy above 2.5 GeV by using a sliding-window algorithm with window size $3 \times 5$ in units of $0.025 \times 0.025$ in $(\eta ,\phi )$ space. From Monte Carlo (MC) simulations of $W$ and $Z$ leptonic decays, the efficiency of the initial cluster reconstruction is expected to be approximately 97 % at $E_{\mathrm {T}}=7{\mathrm {\ GeV}}$ and almost 100 % for electrons with $E_{\mathrm {T}}>20{\mathrm {\ GeV}}$.

*Track association with the cluster:* Within the tracking volume, tracks with $p_{\mathrm {T}}>0.5$ GeV are extrapolated from their last measured point to the middle layer of the EM calorimeter. The extrapolated $\eta$ and $\phi$ coordinates of the impact point are compared to a corresponding seed cluster position in that layer. A track and a cluster are considered to be successfully matched if the distance between the track impact point and the EM cluster barycentre is $|\varDelta \eta | < 0.05$. To account for the effect of bremsstrahlung losses on the azimuthal distance, the size of the $\varDelta \phi$ track–cluster matching window is 0.1 on the side where the extrapolated track bends as it traverses the solenoidal magnetic field. An electron candidate is considered to be reconstructed if at least one track is matched to the seed cluster. In the case where more than one track is matched to a cluster, tracks with hits in the pixel detector or the SCT are given priority, and the match with the smallest $\varDelta R=\sqrt{(\varDelta \eta )^2+(\varDelta \phi )^2}$ distance is chosen. In the absence of a matching track, the cluster is classified as an unconverted photon candidate. Electrons are distinguished from converted photons by investigating the presence of pairs of close-by tracks originating from a vertex displaced from the interaction point and by verifying the location of the first hits along the path of the single tracks [10].

*Reconstructed electron candidate:* After a successful track–cluster matching, the cluster sizes are optimised to take into account the overall energy distributions in the different regions of the calorimeter. In the EM barrel region, the energy of the electron cluster is collected by enlarging its size to $3 \times 7$ in units of $0.025 \times 0.025$ in ($\eta ,\phi$) space, while in the EM endcaps the size is increased to $5 \times 5$. The total reconstructed electron-candidate energy is determined from the sum of four contributions [11]: the estimated energy deposit in the material in front of the EM calorimeter; the measured energy deposit in the cluster, corrected for the estimated fraction of energy measured by the sampling calorimeter; the estimated energy deposit outside the cluster (lateral leakage); and the estimated energy deposit beyond the EM calorimeter (longitudinal leakage). The correction for the material is aided by the measured presampler signal, while the other three corrections are derived from MC simulations. The $(\eta ,\phi )$ spatial coordinates of the electron candidate are taken from the parameters of the matched track at the interaction vertex. The absolute energy scale and the intercalibration of the different parts of the EM calorimeter are determined using tightly selected electrons from $Z \rightarrow ee$, $J/\psi \rightarrow ee$ and $W \rightarrow e\nu$ decays [7].

**Fig. 2:**
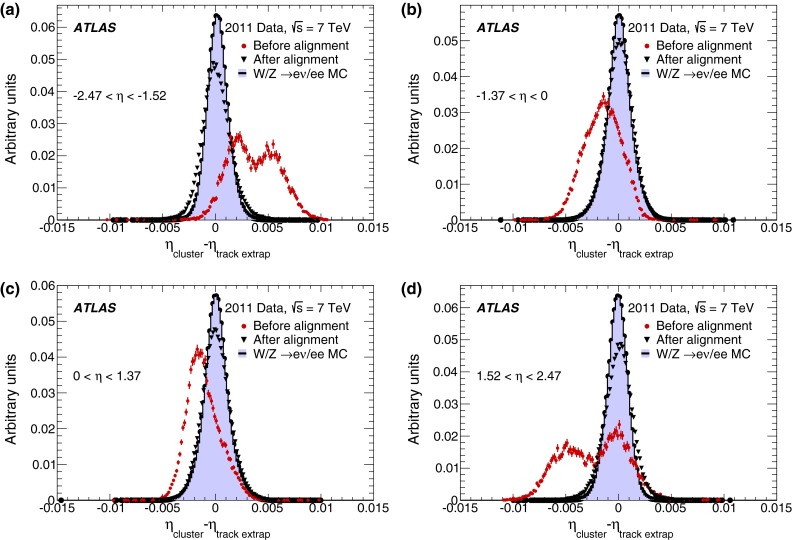
Distributions of the difference between the cluster $\eta$ position determined from the first layer of the EM calorimeter, and the $\eta$ position of the ID track extrapolated to the entrance of that layer. Before the alignment procedure, the estimated detector positions were based on the best knowledge from survey and construction. The distribution is shown before (*red points*) and after (*black triangles*) the alignment corrections. Monte Carlo distributions using a perfect tracker–calorimeter alignment are also shown as a coloured histogram. The four figures correspond to two half-barrels ($-1.37<\eta <0$ in **b** and $0<\eta <1.37$ in **c**) and the two endcaps ($-2.47<\eta <-1.52$ in **a** and $1.52<\eta <2.47$ in **d**). The two-peak structure visible in the endcap plots **a** and **d** before alignment is due to an endcap transverse displacement of 5 mm with respect to the beam-line

The relative alignment of the calorimeter components with respect to the inner detector has been measured using electron candidates with transverse energy $E_{\mathrm {T}}>20$ GeV selected with strict identification criteria, similar to those used for the energy calibration, and compatible with coming from the decay of $W$ or $Z$ bosons. The difference between the electron cluster position and the impact point of the track extrapolation to the calorimeter indicates the size of possible relative displacements between the two detectors. The derived alignment constants are applied to correct both the $\eta$ (as shown in Fig. 2) and $\phi$ electron cluster coordinates.

#### Forward electrons

In the forward region ($2.5<|\eta |\!<\!4.9$), which is not equipped with tracking detectors, the electron reconstruction uses only the information from the EMEC and forward calorimeters and therefore no distinction is possible between electrons and photons. Due to the reduced detector information in this region, the use of forward electrons in physics analyses is restricted to the range $E_{\mathrm {T}}>20$ GeV. In contrast to the fixed-size sliding-window clustering used in the central region, the forward region uses a topological clustering algorithm [12]: cells with deposited energy significantly above the noise level are grouped in three dimensions in an iterative procedure, starting from seed cells. The number of cells in the cluster is not fixed and the sum of their energies defines the energy of the cluster, with corrections made to account for energy losses in the passive material in front of the calorimeters. As determined from simulation, the efficiency of the cluster reconstruction is better than 99 % for $E_{\mathrm {T}}>20$ GeV. An electron candidate in the forward region is reconstructed if it has a transverse energy of $E_{\mathrm {T}}>5$ GeV and has only a small energy component in the hadronic calorimeters. The direction of the forward-electron candidates is defined by the barycentre of the cells belonging to the cluster.

### Electron identification

#### Central electrons

The identification criteria for central-electron candidates are implemented based on sequential cuts on calorimeter, on tracking, and on combined track–cluster variables. These requirements are optimised in 10 cluster-$\eta$ bins, motivated by the structure of the detector, and 11 $E_{\mathrm {T}}$ bins (from 5 to 80 GeV), in order to provide good separation between signal (isolated) electrons and background from hadrons misidentified as electrons, non-isolated electrons (e.g. from semileptonic decays of heavy-flavour particles), and electrons from photon conversions.

Three sets of reference selection criteria, labelled *loose*, *medium* and *tight*, are designed for use in analyses. These three sets were revisited with respect to those described in Ref. [7], which were designed mostly for robustness at the startup of the LHC machine with low-luminosity conditions. These criteria are designed in a hierarchical way so as to provide increasing background-rejection power at some cost to the identification efficiency. The increased background-rejection power was obtained both by adding discriminating variables at each step and by tightening the requirements on the original variables. The different selections used for central-electron identification are detailed in Table 1 and described below.

**Table 1:** Variables used in the *loose*, *medium*, and *tight* electron identification criteria in the central region of the detector ($|\eta |<2.47$)

Category	Description	Variable
***loose***		
Acceptance	$|\eta |<2.47$	
Hadronic leakage	In $|\eta | <0.8$ and $|\eta | >1.37$: ratio of $E_{\mathrm {T}}$ in the first layer of the hadronic calorimeter to $E_{\mathrm {T}}$ of the EM cluster	$R_{{\mathrm{had},1}}$
	In $0.8<|\eta |< 1.37$: ratio of $E_{\mathrm {T}}$ in whole hadronic calorimeter to $E_{\mathrm {T}}$ of the EM cluster	$R_\mathrm{{had}}$
Middle layer of the EM	Ratio of energies in $3 \times 7$ cells over $7 \times 7$ cells	$R_{\eta }$
	Lateral width of the shower	$w_{\eta 2}$
Front layer of the EM	Total shower width	$w_\mathrm{stot}$
	Energy difference of the largest and second largest energy deposits in the cluster divided by their sum	$E_\mathrm{{ratio}}$
Track quality and track–cluster matching	Number of hits in the pixel detector (${>}0$)	
	Number of hits in the silicon detectors (${\ge }7$)	
	$|\varDelta \eta |$ between the cluster position in the first layer and the extrapolated track (${<}0.015$)	$\varDelta \eta _1$
***medium*** **(includes** ***loose*** **with tighter requirements on shower shapes)**		
Track quality and track–cluster matching	Number of hits in the b-layer ${>}0$ for $|\eta |<2.01$	
	Number of hits in the pixel detector ${>}1$ for $|\eta |>2.01$	
	Transverse impact parameter $|d_{0}|<5$ mm	$d_0$
	Tighter $|\varDelta \eta _1|$ cut (${<}0.005$)	
TRT	Loose cut on TRT high-threshold fraction	
***tight*** **(includes** ***medium*** **)**		
Track quality and track–cluster matching	Tighter transverse impact parameter cut ($|d_0|<1$ mm)	
	Asymmetric cut on $\varDelta \phi$ between the cluster position in the middle layer and the extrapolated track	$\varDelta \phi$
	Ratio of the cluster energy to the track momentum	$$E/p $$
TRT	Total number of hits in the TRT	
	Tighter cut on the TRT high-threshold fraction	
Conversions	Reject electron candidates matched to reconstructed photon conversions	

*Loose:* The *loose* selection uses shower-shape variables in both the first and second layers of the EM calorimeter, in contrast to the original selection [7], which did not use the former. As before, hadronic leakage information is used. Additional requirements on the quality of the electron track and track–cluster matching improve the rejection of hadronic backgrounds by a factor of ${\sim }5$ in the $E_{\mathrm {T}}$ range 30 to 40 GeV while maintaining a high identification efficiency.

*Medium:* The *medium* selection adds to the *loose* discriminating variables by requiring the presence of a measured hit in the innermost layer of the pixel detector (to reject electrons from photon conversions), applying a loose selection requirement on the transverse impact parameter $|d_{0}|$, and identifying transition radiation in the TRT (to reject charged-hadron background), when available. The requirements on the discriminating variables in common with the *loose* selection are also tightened, allowing the background-rejection power to increase by approximately an order of magnitude with respect to *loose*.

*Tight:* The *tight* selection makes full use of the particle-identification tools available for electron identification. In addition to the generally tighter requirements on *medium* selection discriminating variables, stricter requirements on track quality in the presence of a track extension in the TRT detector, on the ratio of the EM cluster energy to the track momentum, and a veto on reconstructed photon conversion vertices associated with the cluster [10] are applied. Overall, a rejection power higher by a factor of two is achieved with respect to the *medium* selection.

The *loose*, *medium*, and *tight* identification criteria naturally exclude a large fraction of candidates with additional close-by activity, such as electrons within jets. It is important to note that none of the electron identification criteria explicitly apply requirements on the presence of other particles (additional tracks or energy deposits outside the EM cluster) close to the identified electrons. The optimisation of such dedicated requirements (so-called *isolation* requirements), is strongly dependent on the physics process and is performed separately in each analysis.

#### Forward electrons

Electron identification in the forward region also is based on sequential cuts on discriminating variables; however, these variables are mostly based on topological cluster moments,^3^ as defined in Table 2. As for the central region, three reference sets of selection criteria, labelled *loose*, *medium*, and *tight*, are defined. To compensate for the absence of tracking information in the forward region, variables describing both the lateral and longitudinal shower development are employed. In addition, due to the significantly harsher pile-up conditions at high pseudorapidity with respect to those described in Ref. [7], the identification criteria for forward electrons were redesigned and optimised directly with data in nine cluster-$\eta$ bins: six in the EMEC calorimeter ($2.5<|\eta |<3.16$) and three in the FCal ($3.35<|\eta |<4.90$). The transition region between the two calorimeters ($3.16<|\eta |<3.35$) is excluded from the study. No explicit dependence on cluster $E_{\mathrm {T}}$ or isolation energy is introduced in the forward-electron identification criteria. However, in contrast to the central electrons, the identification criteria are also optimised in four bins of the number of primary vertices reconstructed in the event $N_{\mathrm{PV}}$ (1–3, 4–6, 7–10, $>$10), allowing for similar electron-identification efficiency for different pile-up conditions. These three reference sets use the same variables in each set, but with increasing background rejection power coming from tightened requirements, with the *tight* identification providing a rejection factor approximately two to three times higher than the *loose* selection.

**Table 2:** Variables used to identify electrons in the forward region of the detector ($2.5<|\eta |<4.9$)

Category	Description	Variable
Acceptance	$2.5<|\eta |<4.9$	
Shower depth	Distance of the shower barycentre from the calorimeter front face measured along the shower axis	$\lambda _\mathrm{{centre}}$
Maximum cell energy	Fraction of cluster energy in the most energetic cell	$f_\mathrm{{max}}$
Longitudinal second moment	Second moment of the distance of each cell to the shower centre in the longitudinal direction ($\lambda _i$)	$\langle \lambda ^2 \rangle$
Transverse second moment	Second moment of the distance of each cell to the shower centre in the transverse direction ($r_i$)	$\langle r^2 \rangle$
Normalised lateral moment	$w_2$ and $w_\mathrm{{max}}$ are second moments of $r_i$ for different weights per cell	$\frac{w_2}{w_2+w_\mathrm{{max}}}$
Normalised longitudinal moment	$l_2$ and $l_\mathrm{{max}}$ are the second moments of $\lambda _i$ for different weights per cell	$\frac{l_2}{l_2+l_\mathrm{{max}}}$

### Bremsstrahlung-mitigation algorithms

An electron can lose a significant amount of its energy due to bremsstrahlung when interacting with the material it traverses. Because of the electron’s small mass, radiative losses can be substantial, resulting in alterations of the curvature of the electron’s trajectory when it propagates through a magnetic field and hence of the reconstructed electron track. The electron-reconstruction scheme described in Sect. 3.2.1 employs the same tracking algorithm for all charged particles, with all tracks fitted using a pion mass hypothesis to estimate the material effects. The lack of special treatment for bremsstrahlung effects results in inefficiencies in reconstructing the electron trajectory. It also results in the degradation of the estimated track parameters, increasing with the amount of material encountered. The effect is strongly dependent on the electron pseudorapidity, as shown in Fig. 1. By taking into account possible bremsstrahlung losses (and the resulting alteration of the track curvature), the estimated electron track parameters can be improved. In 2011–2012, a two-step programme was underway in ATLAS to improve electron reconstruction: first to correct all track parameters associated with electron candidates by performing a bremsstrahlung refitting procedure prior to the matching with the electron cluster, and then performing bremsstrahlung recovery at the initial step of the electron trajectory formation, to allow more efficient track reconstruction. By the end of the 2011 data-taking period, the first step [13] was made available to analyses, improving the track-related electron identification variables. The second step was implemented in time for the 2012 data-taking period, increasing the electron reconstruction efficiency by several percent, especially at low $E_{\mathrm {T}}$. Results presented in this paper do not use the bremsstrahlung-mitigation algorithms.

## Methodology for efficiency measurements

Isolated electrons are important ingredients in Standard Model measurements and searches for physics beyond the Standard Model. However, the experimentally determined electron spectra must be corrected for instrumentation inefficiencies, such as those related to trigger, reconstruction, and identification, before absolute measurements can be made. These inefficiencies may be directly estimated from data using so-called tag-and-probe methods [7]. These methods are used to select, from known resonances such as $Z \rightarrow ee$, unbiased samples of electrons (*probes*) by using strict selection requirements on the second object produced from the particle’s decay (*tags*). The efficiency of a requirement can then be determined by applying it directly to the probe sample after accounting for residual background contamination. The efficiency factor relating a true single-electron spectrum to one determined experimentally may be factorised as a product of different efficiency terms:

$$\begin{aligned} \epsilon _\mathrm{e} = \epsilon _\mathrm{cluster}\cdot \epsilon _\mathrm{reco}\cdot \epsilon _\mathrm{id}\cdot \epsilon _\mathrm{trig}\cdot \epsilon _\mathrm{other}, \end{aligned}$$

where $\epsilon _\mathrm{cluster}$ is the efficiency to reconstruct an electromagnetic cluster, $\epsilon _\mathrm{reco}$ is the electron reconstruction algorithm efficiency given the presence of the cluster (Sect. 3.2), and $\epsilon _\mathrm{id}$ is the efficiency of identification criteria with respect to the reconstructed electron candidates (Sect. 3.3). The variable $\epsilon _\mathrm{trig}$ denotes the trigger efficiency with respect to reconstructed electron candidates passing the identification criteria. The variable $\epsilon _\mathrm{other}$ is the efficiency of any extra selection requirements applied to the electrons satisfying the identification criteria, such as isolation of the electron cluster and/or track, or selections on the significance of the impact parameter of the fitted electron track (both are used in many analyses). This paper reports on the measurement of the reconstruction efficiency $\epsilon _\mathrm{reco}$ and the identification efficiency $\epsilon _\mathrm{id}$ as determined from data and compared with expectations from simulated events. The term $\epsilon _\mathrm{cluster}$ is determined from simulation to be close to unity, with typical values in the central and forward regions provided in Sect. 3.2. Measurements of the trigger efficiency $\epsilon _\mathrm{trig}$ can be found in Ref. [14]. The term $\epsilon _\mathrm{other}$ is largely process-dependent and so must be measured separately in each analysis. Section 8 presents a measurement of the efficiency to correctly identify the charge of an electron, $\epsilon _\mathrm{charge}$, with respect to the reconstructed electron candidates satisfying the various identification criteria.

Tag-and-probe-based measurements based on samples of $Z \rightarrow ee$, $W \rightarrow e\nu$, and $J/\psi \rightarrow ee$ events are presented. The combination of the three samples allows efficiency measurements over a significant $E_{\mathrm {T}}$ range, from 7 to 50 GeV, while still providing overlapping measurements between the samples.^4^ In the case of $Z \rightarrow ee$ and $J/\psi \rightarrow ee$ decays, events are selected on the basis of the electron-positron invariant mass and strict identification criteria applied to the tag electron. Electron identification efficiencies are also extracted from $W \rightarrow e\nu$ decays, tagging on the presence of missing transverse momentum in the event; this channel contributes significantly to the overall efficiency determination due to its high statistical power. At the LHC, $J/\psi$ mesons are produced directly and in $b$-hadron decays. Prompt $J/\psi$ decays occur in the vicinity of the primary event vertex while many of the non-prompt $J/\psi$ particles have displaced decay vertices due to the relatively long lifetime of their $b$-hadron parent. The $J/\psi$ candidates come from a mixture of these two processes; however, their ability to extend the reach of efficiency measurements to low $E_{\mathrm {T}}$ makes them nonetheless very attractive, in spite of this added complication.

The shower profiles of electrons in the calorimeters depend on both the energy of the electrons and the amount of material traversed by the electrons before reaching the calorimeter. For this reason, electron efficiency measurements in the central region ($|\eta |<2.47$) are made binned in two dimensions, both transverse energy and pseudorapidity, in contrast to the previous results [7] whose statistical precision could only provide one-dimensional binning in either variable. Eight bins of 5 GeV in transverse energy are used in the range from 10 to 50 GeV, with an additional bin covering the low $E_{\mathrm {T}}$ range from 7 to 10 GeV. Depending on the available statistics in each $E_{\mathrm {T}}$ bin, efficiencies are measured in three different, largely detector-motivated, $\eta$ granularities:

- *coarse:* 11 bins in $\eta$ with limits $-2.47$, $-2.01$, $-1.52$, $-1.37$, $-0.8$, $-0.1$, $0.1$, $0.8$, $1.37$, $1.52$, $2.01$, $2.47$
- *middle:* 20 bins in $\eta$ with $|\eta |$ limits $0.0$, $0.1$, $0.6$, $0.8$, $1.15$, $1.37$, $1.52$, $1.81$, $2.01$, $2.37$, $2.47$
- *fine:* 50 bins in $\eta$ with a typical granularity of 0.1 covering the full pseudorapidity range ($|\eta |<2.47$).

In the forward region the measurements are performed binned only in absolute electron pseudorapidity:

- *forward:* 9 bins in $|\eta |$ with limits $2.5$, $2.6$, $2.7, 2.8$, $2.9$, $3.0$, $3.16$, $3.35$, $3.6$, $4.0$, $4.9$.

The efficiency is defined as the fraction of electrons passing a particular selection in a given ($E_{\mathrm {T}},\eta$) bin. For the case of $\epsilon _\mathrm{reco}$, the electron reconstruction efficiency is calculated with respect to the sample satisfying the cluster-building step. Hence, clusters associated with reconstructed photons are also included in the denominator of the measured reconstruction efficiency, provided that they are separated by $\varDelta R>0.4$ from any other cluster associated with a reconstructed electron. As no reconstructed charge is available for clusters without an associated track, no requirement on the charge of the tag and the probe is applied. For the case of $\epsilon _\mathrm{id}$, the efficiency to identify an electron as *loose*, *medium*, or *tight* is calculated with respect to a reconstructed electron candidate, resulting in three ratios: $\epsilon _\mathrm{loose}$, $\epsilon _\mathrm{medium}$, and $\epsilon _\mathrm{tight}$, respectively. For the case of $\epsilon _\mathrm{charge}$, the efficiency to correctly identify the charge of an electron is calculated by comparing the ensemble of di-electron pairs without any requirement on the sign of the charge of the track to that of the yield of opposite-sign pairs consistent with the decay of a $Z$ boson. The statistical uncertainty of these efficiencies is computed assuming a binomial distribution. If the evaluation of the number of events (before or after the selection under investigation) is the result of a background subtraction, the corresponding uncertainties are also included in the statistical uncertainty.

## The 2011 data and simulation samples

The data recorded during the 2011 proton–proton collision run at 7 TeV are subdivided into several periods corresponding to the changing conditions of the detector, including the energy thresholds of the primary triggers, as well as the instantaneous luminosity of the LHC. Monte Carlo samples are generated to mimic the same period granularity. In order to reproduce the pile-up effects observed in the data, additional inelastic proton–proton interactions in the form of simulated Pythia [16] minimum-bias events are included in the Monte Carlo simulation.

### Samples

All data collected by the ATLAS detector undergo careful scrutiny to ensure the quality of the recorded information. In particular, data used for the efficiency measurements are filtered requiring that all detector subsystems needed in the analysis (calorimeters and tracking detectors) are operating nominally. Several detector defects had minor impacts on the quality of the 2011 data set. The total integrated luminosity used for the measurement presented in this paper is ${\mathcal L}=4.7$ fb$^{-1}$ [17].

Samples of simulated $Z \rightarrow ee$, $W \rightarrow e\nu$, and $J/\psi \rightarrow ee$ decays are used to benchmark the expected electron reconstruction and identification performance. The primary $Z \rightarrow ee$ and $W \rightarrow e\nu$ MC samples are generated with Powheg version r1556 [18–21] and parton showering is accomplished using Pythia version 6.425. The $J/\psi$ samples are generated using the same version of Pythia. All generators are interfaced to Photos version 3.0 [22] to simulate the effect of final-state QED radiation. The generated event samples are passed through a detailed ATLAS detector simulation [23] using GEANT4 [24]. The MC events are reconstructed using the same software suite as used for the data. Because background subtraction is not performed on the MC signal samples when assessing the expected electron efficiency, generator-level information is used to select electrons originating only from $Z \rightarrow ee$, $W \rightarrow e\nu$, or $J/\psi \rightarrow ee$ decays. Correction factors are applied to the simulation to account for known discrepancies with the data. These include corrections in the form of event weights applied to the simulated events to match the average interaction rate per bunch crossing and the width of the beam-spot in the $z$-direction, both as measured in the 2011 data set. Both corrections are important for the measurements presented in this paper since the identification efficiency depends on the instantaneous luminosity and the position of the primary interaction.

**Fig. 3:**
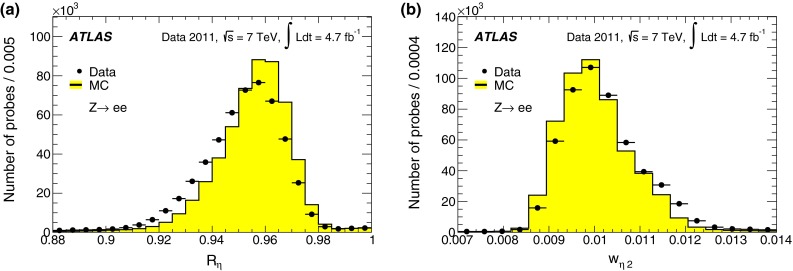
Comparison of the shapes in data and MC simulation for two variables related to the lateral shower extension in the second layer of the EM calorimeter (see Table 1): $R_{\eta }$ in **a** and $w_{\eta 2}$ in **b**. Electrons with $E_{\mathrm {T}}$ in the range 40–45 GeV from $Z \rightarrow ee$ decays are used to extract these shapes

Important improvements to the ATLAS GEANT4 simulation were made as a consequence of observed Monte Carlo–data discrepancies in 2010 related to the transverse shower shapes of electrons in the EM calorimeter [7]. The implementation of a new GEANT4 version (4.9.3), combined with a change of the ATLAS geometry description resulted in a significant improvement in the 2011 MC simulation samples. The residual differences that are still observed when comparing data and MC for some variables, as shown in Fig. 3, have to be taken into account in the analyses by applying appropriate data-to-MC efficiency corrections as presented in this paper.

### Triggers

The samples used in these measurements were selected by the primary electron triggers as well as by specifically designed supporting triggers. In order to keep the trigger rates to an acceptable level with the increase of the instantaneous luminosity in 2011, the primary single-electron trigger selection had to be adjusted several times by raising the minimum transverse energy threshold and tightening the selection criteria. These same trigger conditions are also implemented in the Monte Carlo simulations.

- $Z \rightarrow ee$ events were collected using the unprescaled single-electron triggers, requiring the candidates to pass a minimum $E_{\mathrm {T}}$ threshold. These events were also required to satisfy strict quality criteria; initially, the so-called *medium* and later *medium1* criteria introduced to tighten the requirements on the shower shapes and track properties, limitations on the amount of energy deposited in the hadronic calorimeter, and $\eta$-dependent $E_{\mathrm {T}}$ thresholds (indicated in the trigger name by “vh”) at L1. These triggers are summarised in Table 3 [14].

**Table 3:** Single-electron trigger evolution during the 2011 data taking, with their respective $E_{\mathrm {T}}$ thresholds at EF level

Single-electron	Luminosity	$E_{\mathrm {T}}$ threshold
triggers	$[\mathrm{cm}^{-2}\,\mathrm{s}^{-1}]$	[GeV]
e20_medium	Up to $2\times 10^{33}$	20
e22_medium	$2{-}2.3\times 10^{33}$	22
e22vh_medium1	${>}2.3\times 10^{33}$	22
- $W \rightarrow e\nu$ events were collected with specialised triggers based on the missing transverse momentum^5^ $E_{\mathrm {T}}^{\mathrm {miss}}$ significance $x_{s}=E_{\mathrm {T}}^{\mathrm {miss}}/ ( \alpha ( \sqrt{\sum {E_{\mathrm {T}}}-c}) )$, where the sum runs over all energy deposits and the constants $\alpha$ and $c$ are optimised such that the denominator represents the $E_{\mathrm {T}}^{\mathrm {miss}}$ resolution. The $x_{s}$ variable offers the ability to suppress the background significantly, allowing the triggers to run unprescaled at any pile-up rate. An $x_{s}$ selection requirement was used in combination with an electron $E_{\mathrm {T}}$ cluster threshold of 10 or 13 GeV. During the 2011 run, additional track-quality requirements were applied to the probe electron candidates. The $E_{\mathrm {T}}^{\mathrm {miss}}$ vector was required to be separated by at least $\varDelta \phi =0.7$ from any jet with $p_{\mathrm {T}}>10$ GeV, where the jets were reconstructed with the anti-$k_t$ algorithm [25] with distance parameter $R=0.4$.
- $J/\psi \rightarrow ee$ events were collected with five dedicated prescaled di-electron triggers, mainly enabled towards the end of LHC fills, by requiring a candidate with *tight* identification criteria exceeding a minimum $E_{\mathrm {T}}$ threshold for the tag electron, an electromagnetic cluster exceeding a minimum $E_{\mathrm {T}}$ threshold for the probe electron, and a tag–probe invariant mass between 1 and 6 GeV. These triggers are summarised in Table 4.

**Table 4:** Di-electron triggers used for collecting $J/\psi \rightarrow ee$ events. The first part of each trigger name indicates the threshold of the tight tag electron, while the second corresponds to the loosely selected probe one. The di-electron mass is required to be in the 1–6 GeV mass range

Di-electron	Tag electron $E_{\mathrm {T}}$	Probe electron $E_{\mathrm {T}}$
triggers	threshold [GeV]	threshold [GeV]
e5e4	5	4
e5e9	5	9
e5e14	5	14
e9e4	9	4
e14e4	14	4

While the triggers used for the collection of $W \rightarrow e\nu$ and $J/\psi \rightarrow ee$ events do apply some requirements on probe electrons and on the event topology, these are chosen to be looser than the offline selection and thus do not impact the efficiency measurement. In the case of $Z \rightarrow ee$ collection, it is ensured that the tag electron was sufficient to trigger the event, thus avoiding any bias on the probe properties.

## Identification efficiency measurement

### Central-electron identification efficiency

Events from $W \rightarrow e\nu$, $Z \rightarrow ee$, and $J/\psi \rightarrow ee$ samples are used to measure the central-electron identification efficiencies for various identification criteria, in the transverse energy range from 7 to 50 GeV and pseudorapidity range $|\eta |$ $<2.47$.

#### Selection requirements and sample sizes

A common set of requirements is applied to all triggered events to ensure good data quality and suppress contamination from background events. All electron candidates, whether they be tag or probe electrons, must be reconstructed within $|\eta |$ $<2.47$ with at least six hits in the SCT and one in the pixel detector. The effect of these requirements is accounted for in the reconstruction efficiency; see Sect. 7. Tight selection criteria are applied to the tagging object that triggered the event, that is, to one of the two electrons in $Z \rightarrow ee$ and $J/\psi \rightarrow ee$ events or to $E_{\mathrm {T}}^{\mathrm {miss}}$ in the case of $W \rightarrow e\nu$ events. For the case of $W \rightarrow e\nu$ and $Z \rightarrow ee$ candidates, the probe electrons must also satisfy a requirement limiting the amount of leakage of the shower into the hadronic calorimeter (also accounted for in the reconstruction efficiency; see Sect. 7). Further criteria are imposed in each channel to improve the separation between signal and background events.

$W \rightarrow e\nu$ *channel:* A range of requirements is applied to the minimum value of the transverse mass^6^ $m_\mathrm{T}$ (40 to 50 GeV), and on the missing transverse momentum, $E_{\mathrm {T}}^{\mathrm {miss}}$ (25 to 40 GeV), of the event in order to obtain event samples with differing background fractions. A minimum transverse-energy requirement of $E_{\mathrm {T}}>15$ GeV is applied to the probe electrons and the entire event is discarded if more than one probe candidate in a given event satisfies the *medium* criteria. Two additional requirements are imposed in order to reduce contributions from hadrons misidentified as electrons. The probe electron candidate is required to be separated from any $R=0.4$ anti-$k_t$ jet with $p_{\mathrm {T}}>25$ GeV found within a cone of radius $\varDelta R = 0.4$. Similarly, the $E_{\mathrm {T}}^{\mathrm {miss}}$ vector must be separated from jets with $p_{\mathrm {T}}> 25$ GeV by at least an angular distance of $\varDelta \phi =0.7$. After the final selection, a sample of 6.8 million $W \rightarrow e\nu$ candidate events was collected when requiring $E_{\mathrm {T}}^{\mathrm {miss}}> 25$ GeV and $m_\mathrm{T}> 40$ GeV.

$Z \rightarrow ee$ *channel:* The tag electron is required to have $E_{\mathrm {T}}>20$ GeV and to lie outside the calorimeter transition region ($1.37<|\eta |<1.52$). The probe electron must have $E_{\mathrm {T}}>15$ GeV and be separated from any jet with $p_{\mathrm {T}}>20$ GeV found within a cone of $\varDelta R = 0.4$. For each pair, the tag and the probe electrons are required to have opposite reconstructed charges. A typical di-electron invariant mass range used in this analysis is 80 to 100 GeV, although this range is varied in systematic studies. After the final selection, a sample of 2.1 million probes from $Z \rightarrow ee$ candidate events with opposite-charge electrons is extracted from the 2011 data set.

**Fig. 4:**
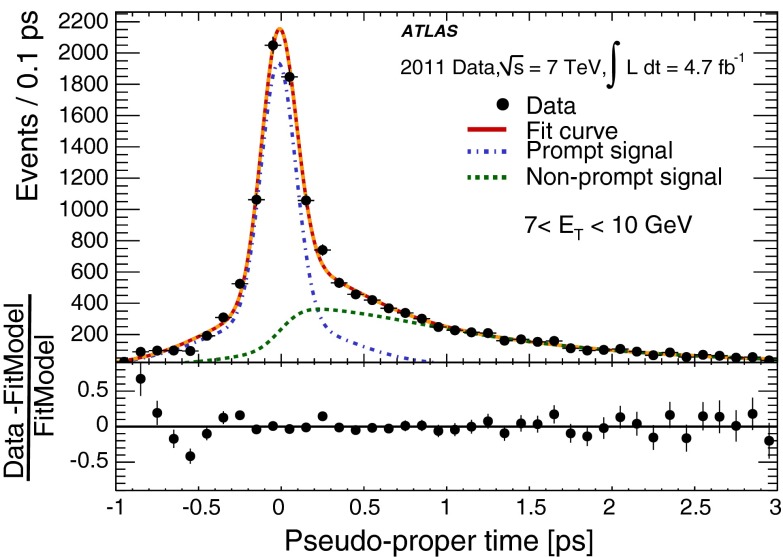
Pseudo-proper time fit of $J/\psi \rightarrow ee$ candidate events for all selected probes within the $E_{\mathrm {T}}$ range 7–10 GeV and integrated over $\eta$. The prompt contribution is modelled by two Gaussian functions, while the non-prompt component uses an exponential function convolved with two Gaussians. Points with *error bars* represent the data sample after background subtraction. The *blue dashed line* shows the prompt signal component while the non-prompt component is drawn with a *dashed green line*. The *red curve* is the sum of the fitted prompt and non-prompt components

$J/\psi \rightarrow ee$ *channel:* The $J/\psi \rightarrow ee$ events come from a mixture of both the prompt and non-prompt decays, with their relative fraction depending both on the triggers used to collect the data and also on the $E_{\mathrm {T}}$ of the probe electrons. Given the difficulties associated with the fact that electrons from non-prompt decays are often surrounded by hadronic activity, two methods have been developed to measure the efficiency for isolated electrons at low $E_{\mathrm {T}}$, both exploiting the pseudo-proper time variable.^7^ The first method, the so-called “*short-lifetime method*” uses $J/\psi \rightarrow ee$ decays measured within very small values of the pseudo-proper time where the prompt component is enhanced, thereby limiting the non-prompt contribution ($f_\mathrm{NP}$) to 8–20 % of the yield. The second method, the so-called “*lifetime-fit method*”, uses the full $J/\psi \rightarrow ee$ candidate sample, corrected for the non-prompt fraction, which is obtained by performing a fit of the pseudo-proper time distribution at each identification stage. An example of this pseudo-proper time fit is shown in Fig. 4. For both $J/\psi \rightarrow ee$ methods, the main challenge is the suppression of the large background present in the low electron $E_{\mathrm {T}}$ region. In order to reduce this background, tighter requirements are imposed on the quantities measured with the TRT hits associated with the tag electron, and the probe electron is required to be isolated from surrounding energy deposits.^8^ Moreover, both the tag and probe tracks are required to originate from the same primary vertex and to be within 0.2 mm of each other in the $z$-direction at the vertex $(x,y)$-position. The probe electron must have $E_{\mathrm {T}}>5$ GeV. Both the tag and probe are permitted to point toward the calorimeter transition region. After the final selection, a sample of 120,000 $J/\psi \rightarrow ee$ candidate events with opposite-charge electrons is collected in the invariant mass range 2.8–3.3 GeV.

**Fig. 5:**
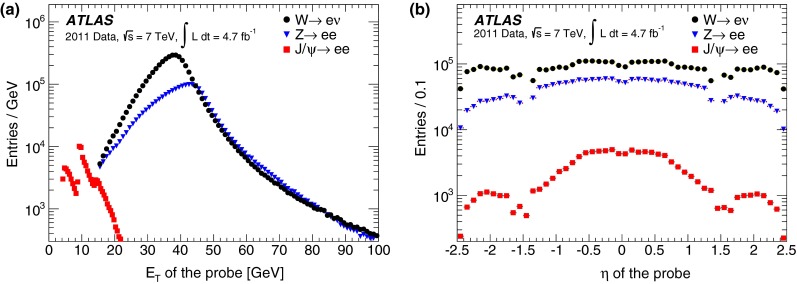
Distributions of probe $E_{\mathrm {T}}$ in **a** and $\eta$ in **b** for the three samples of probes satisfying *tight* identification criteria. The non-continuous $E_{\mathrm {T}}$ spectrum of the $J/\psi \rightarrow ee$ sample is due to the different $E_{\mathrm {T}}$ thresholds of the triggers utilised to collect this sample

The $E_{\mathrm {T}}$ and $\eta$ distributions of *tight* electron probes for the three tag-and-probe samples are shown in Fig. 5.

#### Background evaluation

After the selections described in Sect. 6.1.1 are applied to the data, the three samples still contain background originating from hadrons misidentified as electrons as well as from true electrons from photon conversions and non-isolated electrons originating from heavy-flavour decays. For each sample and in each $(E_{\mathrm {T}},\eta )$ bin, the level of background is evaluated by the use of sensitive discriminating variables to build templates able to provide some separation between signal and background events. These templates are then either fitted or normalised to data to evaluate and subtract the estimated background component in the signal sample.

**Fig. 6:**
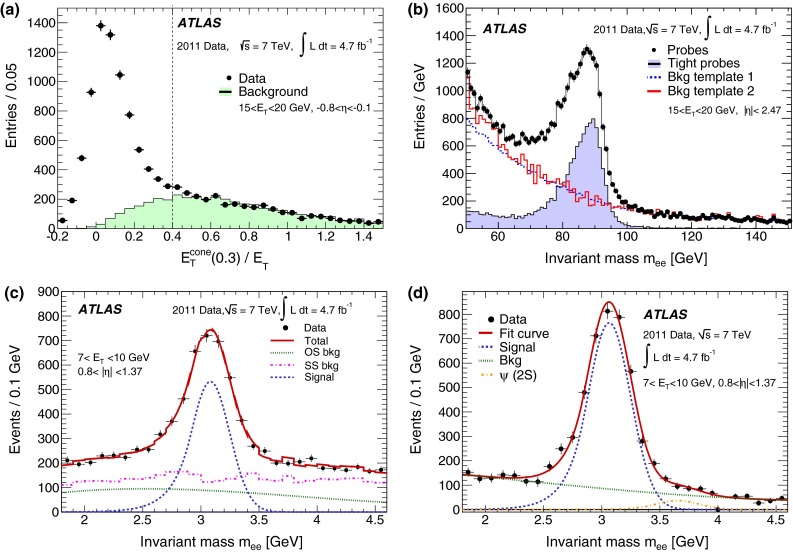
Examples of discriminating variables and background-subtraction techniques for illustrative $(E_{\mathrm {T}},\eta )$ bins. **a** The $E_\mathrm{{T}}^\mathrm{{cone}}(0.3)/E_\mathrm{{T}}$ distribution of probes in the $W \rightarrow e\nu$ sample superimposed with the normalised background template. The *black dashed line* indicates the threshold chosen to delineate the signal and background regions. The $E_\mathrm{{T}}^\mathrm{{cone}}(0.3)/E_\mathrm{{T}}$ variable may take negative values due to the applied average corrections for electronic noise and pileup. **b** Invariant mass distribution in the $Z \rightarrow ee$ sample. The normalised shapes of two different background templates are also shown (see text for details). The invariant mass for pairs where the probe satisfies the *tight* criteria is also shown. **c** Invariant mass distribution for the $J/\psi \rightarrow ee$ sample in the short-lifetime range. The *purple curve* corresponds to the measured background with same-sign (SS) pairs, the *dashed green line* shows the opposite-sign (OS) background, the *blue curve* indicates the extracted signal and the *red line* is the fit to data taking into account signal, background, and $\psi (2S)$ (not shown in the figure) contributions. For presentational purposes the *red line* has been smoothed. **d** Invariant mass distribution for the $J/\psi \rightarrow ee$ sample using the lifetime-fit method. Points with *error bars* represent the number of opposite-sign minus the number of same-sign data pairs, the fitted signal is drawn by the *dashed blue line*, and the $\psi (2S)$ resonance by the *dashed orange line*. The residual opposite-sign background is represented by the *dashed green curve*

$W \rightarrow e\nu$ *channel:* Electron isolation [27] is used as the discriminating variable. Templates are built from the sum of the transverse energies in the electromagnetic and hadronic calorimeters contained in a cone of size $\varDelta R= X$ around the probe, excluding the probe’s contribution. The size $X$ of the cone is typically 0.3 or 0.4. This isolation variable is corrected on an event-by-event basis for pile-up and underlying event contributions [28] and then normalised to the probe’s transverse energy. The resulting quantity is referred to as $E_\mathrm{{T}}^\mathrm{{cone}}(X)/E_\mathrm{{T}}$. The background template is constructed from the probe selection by reversing two of the electron identification criteria, namely the total shower width $w_{\mathrm{stot}}$ and the ratio of high-threshold hits to all TRT hits (see Table 1). To ensure adequate statistics in each bin, the background templates are constructed in ($E_{\mathrm {T}}$,$|\eta |$) bins, assuming similar background at positive and negative pseudorapidity values. In the outermost $|\eta |$ bins where no information from the TRT is available, the template from the last bin with TRT information is employed. A threshold requirement is applied to the $E_\mathrm{{T}}^\mathrm{{cone}}(X)/E_\mathrm{{T}}$ variable to separate the signal-dominated and background-dominated regions located below and above this threshold, respectively. The $E_\mathrm{{T}}^\mathrm{{cone}}(X)/E_\mathrm{{T}}$ spectrum is normalised to the data in this latter region and then used to estimate the background fraction in the signal-dominated region located below the threshold. Figure 6a shows a typical $E_\mathrm{{T}}^\mathrm{{cone}}(0.3)/E_\mathrm{{T}}$ distribution together with the normalised template shape. The signal-to-background ratio S/B typically varies from 6 to 60 for probes with $E_{\mathrm {T}}$ in the ranges of 15–20 to 35–40 GeV, respectively. After performing this background subtraction, 5.2 million events remain in the signal region. As part of the systematic uncertainties studies, templates are also built by applying an additional reverse requirement on $R_{\phi }$ 9 to the original template selection. Both sets of templates adequately describe the high $E_\mathrm{{T}}^\mathrm{{cone}}(X)/E_\mathrm{{T}}$ tail while offering differing shapes close to the signal region.

$Z \rightarrow ee$ *channel:* Two discriminating variables are used to evaluate the background yield in this channel. The first variable is the invariant mass distribution $m_{ee}$ of the tag–probe pair. In this case, the background template is constructed from events failing at least two *loose* identification requirements and having a significant energy deposit in a cone around the probe (see “Bkg template 1” in Fig. 6b). This template is normalised to the invariant mass distribution of reconstructed events in the high-mass region of $m_{ee}>120$ GeV and then used to evaluate the background fraction in the signal region (typically defined as $80< m_{ee}<100$ GeV). A small correction of $\le$1 % is performed to account for $Z/\gamma ^* \rightarrow ee$ signal contribution in the high-mass tail. This is estimated from signal MC normalized to data in the peak region after tight identification cuts. In comparison to using a functional fit to describe the background shape, this method has the advantage of providing reliable results over the entire $(E_{\mathrm {T}},\eta )$ kinematic range. The second variable employed is the $E_\mathrm{{T}}^\mathrm{{cone}}(X)/E_\mathrm{{T}}$ value of the probe, as used in the $W \rightarrow e\nu$ channel and following the same background subtraction techniques. A typical invariant mass distribution is shown in Fig. 6b. The S/B ratio typically varies from 5 to 160 for probes with $E_{\mathrm {T}}$ in the ranges of 15–20 and 35–40 GeV, respectively. After performing this background subtraction, two million probes remain in the signal region.

$J/\psi \rightarrow ee$ *channel:* As for the $Z \rightarrow ee$ channel, the discriminating variable is the tag–probe invariant mass distribution. The $m_{ee}$ spectrum of opposite-sign pairs is fitted, typically in the range of 1.8 to 4.6 GeV, considering four distinct components. Two Crystal Ball functions [29] separately model the signal shape and that of the $\psi (2S)$ resonance (the latter function is centred on the nominal PDG [26] value). The background contribution in the signal region is largely modelled by same-sign pairs as measured in data, with an additional Chebyshev polynomial used to model the remaining background from opposite-sign pairs. For the short-lifetime method, these contributions are fitted to the $m_{ee}$ spectrum as measured in data to evaluate the background contribution in the signal region (see Fig. 6c). For the lifetime-fit method, an unbinned maximum likelihood fit is performed, where same (opposite)-sign pairs are considered with a negative (positive) weight (see Fig. 6d). The $J/\psi \rightarrow ee$ sample suffers from a higher background contamination than the other two channels such that the S/B ratio in the typical signal extraction range of $2.8<m_{ee}<3$ GeV varies between $\sim$0.5 and $\sim$3. After performing the background subtraction, 88,000 (66,000) events remain in the signal region in the full (short) pseudo-proper time range.

#### Identification efficiency measurement systematics

For all three channels, the dominant systematic uncertainties are related to the evaluation of the background contribution to the signal region. Possible biases affecting the efficiency measurement are investigated by varying the selection of events such that the signal-to-background ratio is modified substantially or by re-evaluating the efficiencies with alternative templates or background models. Each analysis is repeated with a large set of variations and the spread of the corresponding results is used to quantify the systematic uncertainties. These variations are designed to allow a reasonable modification of the S/B ratio depending on the background level affecting each mode.

$W \rightarrow e\nu$ *channel:* The baseline sample of $W \rightarrow e\nu$ events is varied by using alternative $E_{\mathrm {T}}^{\mathrm {miss}}$ and $m_{\mathrm{T}}$ selection requirements, and by changing the isolation discriminating variable ($E_\mathrm{{T}}^\mathrm{{cone}}(0.4)/E_\mathrm{{T}}$ and $E_\mathrm{{T}}^\mathrm{{cone}}(0.3)/E_\mathrm{{T}}$) as well as its associated threshold requirement used to delineate the signal and background regions. For each variation, both sets of background templates are used to normalise the isolation distributions above the thresholds. Within the 80 variations used, the S/B ratio distribution in the signal region exhibits an RMS (Root Mean Square) of ${\sim }30$ % at low $E_{\mathrm {T}}$ (15–20 GeV) and ${\sim }25$ % at high $E_{\mathrm {T}}$ (35–40 GeV). The combined effect of the charge misidentification and the different $W^+$ and $W^-$ production cross-sections at the LHC leads to an up to 5 % difference in efficiency using the *tight* criteria between $e^+$ and $e^-$ in the calorimeter endcap bins for probes with $25<$ $E_{\mathrm {T}}$ $<30$ GeV. This difference is very well modelled in the MC efficiency, leading to a negligible uncertainty for most analyses.

**Fig. 7:**
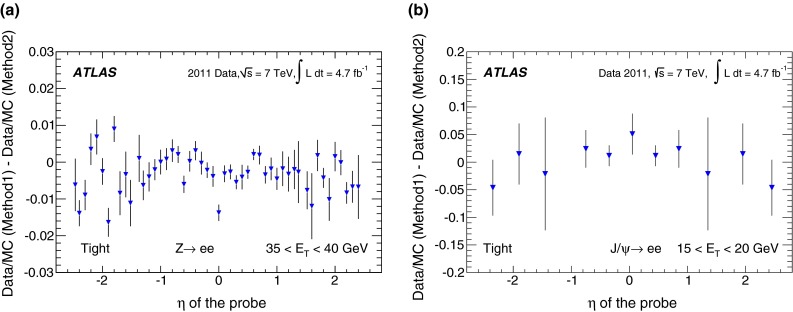
**a** Data-to-MC efficiency ratio difference between the two methods to estimate background (Method 1: invariant mass, Method 2: isolation) used in the $Z \rightarrow ee$ analysis for central electrons, for the *tight* criteria and for probes in the 35–40 GeV $E_{\mathrm {T}}$ bin. **b** The same difference for the lifetime-fit (Method 1) and short-lifetime (Method 2) methods used for the $J/\psi \rightarrow ee$ analysis for *tight* criteria and for probes in 15–20 GeV bin. In both figures, the *error bars* represent only the systematic uncertainties associated with the individual methods

$Z \rightarrow ee$ *channel:* The baseline sample of $Z \rightarrow ee$ events is modified by using alternative selection criteria defining the tag electrons. Three $m_{ee}$ windows (80–100, 70–100 and 75–105 GeV) are used to extract the signal events. Moreover, the size and composition of the background are varied by modifying the reverse requirements used to generate the templates. As an example, the curves “Bkg template 1 and 2” in Fig. 6b are similar in that the events used to build these templates are required to fail some of the *loose* identification requirements (template 1 fails at least two requirements while template 2 fails three) and have a significant energy deposit in a cone around the probe. However, in contrast to template 1, template 2 is also built from events passing additional track-quality requirements and having little hadronic activity associated with the candidate. In the case where the invariant mass is the discriminating variable, an isolation condition ($E_{\mathrm{T}}^\mathrm{{cone}} (0.4) <5$ GeV) is optionally applied to the tag requirement. A total of 36 variations are performed, for which the S/B ratio distribution exhibits an RMS of ${\sim }10$ %. In the case where the isolation of the probe electron plays the role of discriminating variable, the radius of the isolation cone and its associated threshold are also varied, giving in total 120 variations. The method employing the invariant mass as the discriminating variable is used as the primary efficiency measurement. However, the efficiencies computed using either variable agree well with each other within the systematic uncertainties. Figure 7a shows the differences of the data-to-MC *tight* efficiency ratios between the two methods in the $E_{\mathrm {T}}=35{-}40~\hbox {GeV}$ bin, which are generally compatible with zero within less than two standard deviations; these differences are considered as additional uncorrelated systematic uncertainties on the primary measurement.

$J/\psi \rightarrow ee$ *channel:* The baseline sample of $J/\psi \rightarrow ee$ events is similarly modified by using alternative selection criteria to define the tag electron (additional isolation criteria, tight TRT requirements) and by enlarging the 2.8–3.3 GeV mass window defining the signal range. The functional fit for the background from opposite-sign pairs is modified to assess the uncertainty on the background subtraction (using Chebyshev polynomial functions or exponential fits). The range and the function used for the pseudo-proper time fit as well as the size of the isolation cone and its associated threshold are also varied. Both the track-based and energy-based isolation criteria are investigated. A total of 76 and 52 variations resulting in an S/B ratio distribution RMS of ${\sim } 30$ % are used for the lifetime-fit and the short-lifetime methods, respectively.

The method using the short-lifetime range relies on the non-prompt fraction, $f_{\mathrm{NP}}$, extracted from the $J/\psi$ differential cross-section measurement [30], which is used to combine the MC samples corresponding to prompt and non-prompt $J/\psi$ production. Selections targeting further suppression of the non-isolated probes decrease $f_{\mathrm{NP}}$, as expected, and this variation is taken into account as predicted by simulation. The non-prompt fraction increases with the probe $E_{\mathrm {T}}$ and is found to be independent of $\eta$. It enters into the computation of the combined MC efficiency prediction with an uncertainty of 10 %. In contrast, the lifetime-fit method extracts $f_{\mathrm{NP}}$ from the data, by fitting the lifetime distribution in the range from $-1$ to $+3\,\mathrm{ps}$. As in the first method, this fraction is computed in bins of $E_{\mathrm {T}}$ only, since no significant variation was observed as a function of $\eta$. Systematic uncertainties on the value of $f_{\mathrm{NP}}$ obtained from data are assessed by varying the range and the function used in the fit. The results from the two methods agree reasonably well, within the total uncertainties, as shown in Fig. 7b where the difference of the data-to-MC *tight* efficiency ratios between the two methods is shown for the bin $E_{\mathrm {T}}=15{-}20$ GeV. There is an approximate 75 % statistical overlap between the candidates selected by the two methods. In the final combination, both the short-lifetime and lifetime-fit methods are treated as variations of a single measurement.

**Fig. 8:**
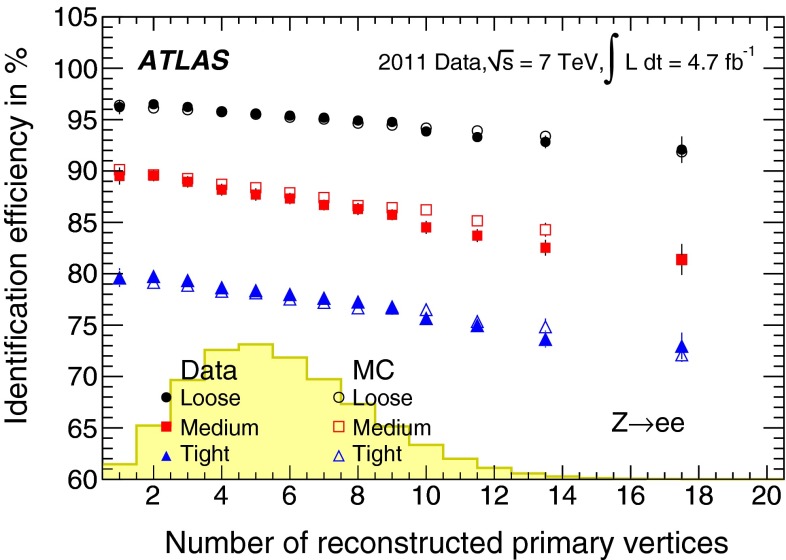
The *loose*, *medium*, and *tight* identification efficiencies as a function of the number of reconstructed primary vertices in the event, for $Z \rightarrow ee$ events and for central-electron probes in the $E_{\mathrm {T}}$ range 15–50 GeV. The quoted *error bars* correspond to the total uncertainties. The observed loss in efficiency is well modelled by the simulation. The *yellow histogram* indicates the $N_\mathrm{{PV}}$ distribution in data

The steady increase of the instantaneous luminosity during the 2011 period induced pile-up effects that varied proportionally to the average number of interactions per beam crossing. Increased pile-up causes higher-energy deposits in the calorimeters and more tracks in the inner detector, which may impact the electron reconstruction and identification. These effects are confirmed when measuring the identification efficiency with $Z \rightarrow ee$ events as a function of the number of reconstructed primary vertices in an event (see Fig. 8), where the efficiency is seen to drop by up to 2 and 5 % for the *loose* and *tight* criteria, respectively. These effects are well modelled by simulation with a maximum difference of approximately two standard deviations observed in the case of *medium* criteria. Variations of the pile-up simulation and of the weighting procedure applied to the simulation to match the pile-up conditions observed in data impact the efficiency at the per mil level.

#### Combination and results

**Fig. 9:**
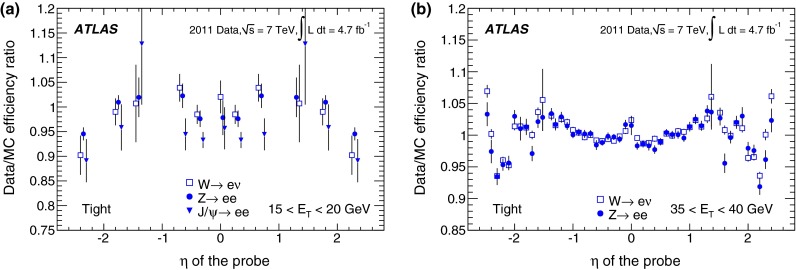
Comparison of the scale factors extracted from the various channels in two $E_{\mathrm {T}}$ bins, shown as a function of the *tight* probe-electron pseudorapidity. In **a**, scale factors from $Z \rightarrow ee$, $W \rightarrow e\nu$, and $J/\psi \rightarrow ee$ are compared in the $E_{\mathrm {T}}$ range 15–20 GeV. In **b**, scale factors from $Z \rightarrow ee$ and $W \rightarrow e\nu$ are shown in the $E_{\mathrm {T}}$ range 35–40 GeV. The *error bars* correspond to the total uncertainties in each channel. Some points are slightly shifted horizontally within the $\eta$ bin for better visibility

The $Z \rightarrow ee$, $W \rightarrow e\nu$, and $J/\psi \rightarrow ee$ channels are statistically independent and so are combined to increase the precision of the identification efficiency measurements. Although the efficiencies in a given $(E_{\mathrm {T}},\eta )$ bin may be slightly different in each channel due to effects related to e.g. resolution and migration effects or the influence of the trigger, these differences are expected largely to cancel when taking the data-to-MC efficiency ratios, referred to as *scale factors* ($\mathrm{SF}$). The combination of the three channels is therefore performed by first calculating the corresponding scale factors in a double-differential binning in electron $E_{\mathrm {T}}$ and $\eta$. As examples, the scale factors of the different channels are shown for two illustrative $E_{\mathrm {T}}$ bins in Fig. 9. The agreement among the channels is in general fair, with the most notable discrepancy observed in the $E_{\mathrm {T}}$ range of 15–20 GeV where the $J/\psi \rightarrow ee$ results in the barrel region are lower than for $Z \rightarrow ee$ and $W \rightarrow e\nu$ with a significance of approximately two standard deviations.

A global $\chi ^{2}$ minimisation [31] is used to compute an average value of the $\mathrm{SF}^{i}$ in each bin $i$ common to all channels:

$$\begin{aligned} \chi ^2&= \sum _{i,k} \frac{\left[ \mu ^{i,k} - \mathrm{SF}^i - \sum _{j}\gamma ^{i,k}_{j}\mathrm{SF}^i b_j\right] ^2}{\left( \delta ^{i,k}_\mathrm{sta}\right) ^2\mu ^{i,k} \mathrm{SF}^i\left( 1 - \sum _{j}\gamma ^{i,k}_{j}b_j\right) + \left( \delta ^{i,k}_\mathrm{unc}\mathrm{SF}^i\right) ^2} \\&+\sum _{j}b^2_j, \end{aligned}$$

where $i$, $k$, and $j$ indices run over the $(E_{\mathrm {T}},\eta )$ bins, the three channels, and the correlated systematics, respectively. The latter are extracted from the systematic variations used to compute the scale factor $\mu ^i_k$ in each channel. The variables $\delta ^{i,k}_\mathrm{sta}$, $\delta ^{i,k}_\mathrm{unc}$, and $\gamma ^{i,k}_{j}$ represent the relative statistical, uncorrelated, and correlated systematic uncertainties, respectively. The nuisance parameters $b_{j}$ are related to correlated uncertainties, which are dominated by the background subtraction uncertainties. The combined scale factors are given by $\mathrm{SF}^i$.

During the minimisation procedure, the central values of the scale factors may be shifted by an amount which is a fraction of the correlated uncertainties, such that the minimal $\chi ^{2}$ is reached. In 0.5 % of all bins, the absolute value of the pull^10^ is larger than two. To be conservative, the uncorrelated uncertainties are in this case inflated by the pull divided by $\sqrt{2}$ and the global minimisation is performed once again. The combination of independent measurements constrains the bin-to-bin correlated uncertainties and reduces their size by up to about 30 %, thereby reducing the total uncertainty. This reduction is most significant in the range $E_{\mathrm {T}}=25{-}40$ GeV, where the $Z \rightarrow ee$ and $W \rightarrow e\nu$ measurements have the highest statistical precision.

*High-* $E_{\mathrm {T}}$ *measurements:* $E_{\mathrm {T}}>20$ GeV. In this region, copious statistics from the low background $Z \rightarrow ee$ and $W \rightarrow e\nu$ channels are available and so the measurement is performed in all three $\eta$ granularities (*coarse, middle, fine*). The total uncertainty in this region is at most 1–2 % for *tight* electrons. In general, the precision reaches the few per mil level at 35 GeV and is statistically limited.

*Low-* $E_{\mathrm {T}}$ *measurements:* $7<E_{\mathrm {T}}<20$ GeV. In this region, the measurement is driven by the $J/\psi \rightarrow ee$ sample, although in the 15–20 GeV bin results from both $W \rightarrow e\nu$ and $Z \rightarrow ee$ are also used in the combination. In this range, only the coarse $\eta$ binning is used due to the statistics available for the measurements. The measurement is limited by the statistical precision and the total uncertainty varies from 3 % in the calorimeter barrel regions to 7 % in the endcap regions.

**Fig. 10:**
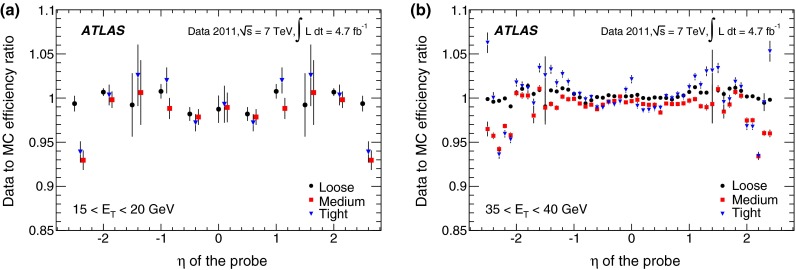
Examples of combined scale factors for the three identification criteria (*loose*, *medium*, *tight*) as a function of the probe-electron pseudorapidity. Results are shown for 15–20 GeV in **a** and 35–40 GeV in **b** probes. In each $\eta$ bin, the points for *loose*, *medium* and *tight* criteria are slightly shifted horizontally for better visibility. The *error bars* indicate the total uncertainties

Figure 10 illustrates some of the combined scale factors at low and high probe-electron $E_{\mathrm {T}}$ resulting from this minimisation procedure. These scale factors are used in all analyses involving electrons, to correct for residual differences between data and simulation that are mainly due to the modelling of the shower shapes in the calorimeter and to the TRT detector calibration in the region $1<|\eta |<2$. These corrections are usually no more than a few percent.

**Fig. 11:**
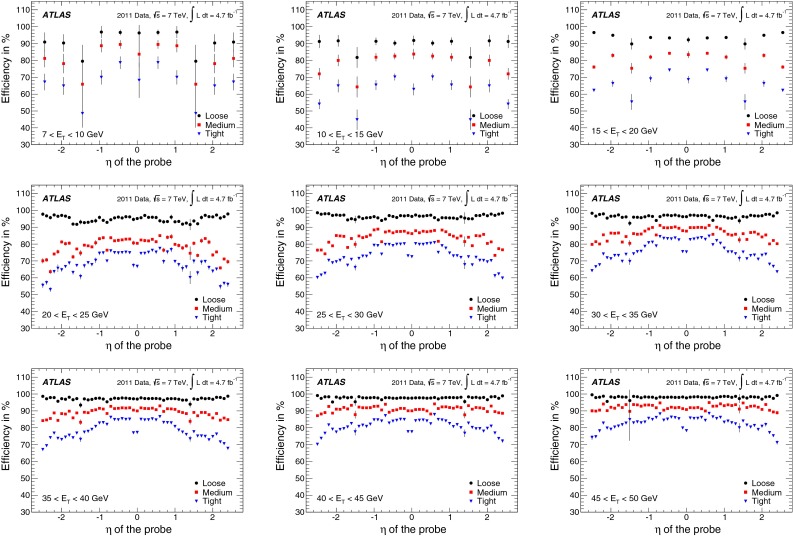
Electron identification efficiencies, extracted by multiplying the combined scale factors evaluated from the $Z \rightarrow ee$, $W \rightarrow e\nu$, and $J/\psi \rightarrow ee$ channels by efficiencies computed from a $Z \rightarrow ee$ Monte Carlo simulation, as a function of the $\eta$ value of the probe for nine $E_{\mathrm {T}}$ bins, from 7–10 GeV (*top*) to 45–50 GeV (*bottom*). The three colours correspond to the three identification criteria (*loose*, *medium*, *tight*). For $E_{\mathrm {T}}<20$ GeV, the coarse binning is used and the efficiencies are plotted symmetrically for both the positive and negative $\eta$ bins. For $E_{\mathrm {T}}>20$ GeV, the efficiencies are shown in the 50 $\eta$ bins available using the fine granularity. The *error bars* indicate the total uncertainties

**Fig. 12:**
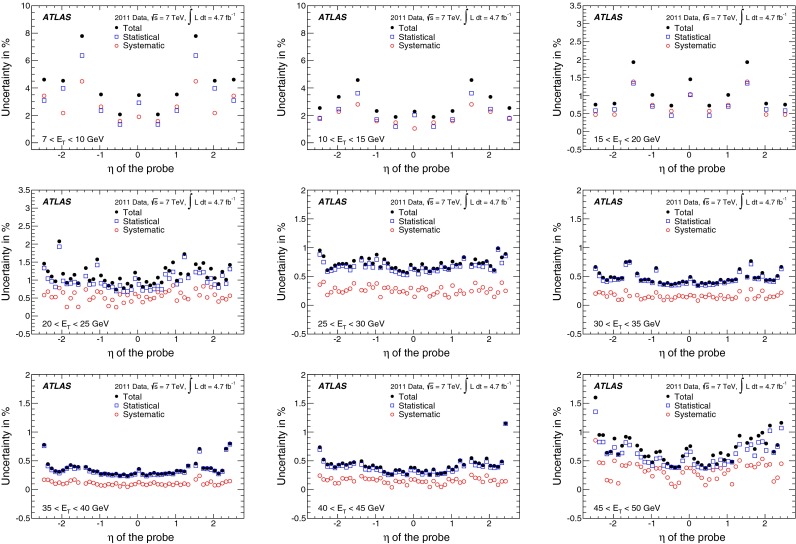
Total, systematic, and statistical uncertainties of the *tight* efficiency (extracted by multiplying the combined scale factors evaluated from the $Z \rightarrow ee$, $W \rightarrow e\nu$, and $J/\psi \rightarrow ee$ channels by efficiencies computed from a $Z \rightarrow ee$ Monte Carlo simulation) as a function of the $\eta$ value of the probe for nine $E_{\mathrm {T}}$ bins, from 7–10 GeV (*top*) to 45–50 GeV (*bottom*). For $E_{\mathrm {T}}<20$ GeV, the coarse binning is used and the uncertainties are plotted symmetrically for both the positive and negative $\eta$ bins. For $E_{\mathrm {T}}>20$ GeV, the uncertainties are shown in the 50 $\eta$ bins available using the fine granularity. The total uncertainties are dominated by the statistical component. The systematic uncertainties are dominated by the uncorrelated component, which is largely due to the difference of the two $Z \rightarrow ee$ methods and thus affected by limited statistics of the different data samples employed for the background-subtraction procedures

In the following, the combined data efficiencies are extracted by multiplying the combined scale factors by the efficiencies computed from a $Z \rightarrow ee$ Monte Carlo simulation. Figure 11 shows, for the coarse $\eta$ granularity in the low-$E_{\mathrm {T}}$ region and the fine granularity in the region $E_{\mathrm {T}}>20$ GeV, the efficiencies obtained in this way. The precision of the efficiency measurements is in general dominated by the statistical component, as shown in Fig. 12 for the *tight* criteria. Possible sources of systematic uncertainties arising from the choice of MC generator to derive the scale factors are not accounted for in this analysis but are expected to have a negligible impact on these results. This is due to the fact that the final results as shown in Figs. 11 and 12 are obtained from data-driven efficiency measurements, combined through the use of scale factors, but then multiplied by a $Z \rightarrow ee$ MC sample.

In the case of *loose* identification criteria, efficiencies are fairly uniform with pseudorapidity, while a slight dependence is observed both for *medium* and *tight*. The identification efficiency is sensitive to the readout granularity of the detectors and to the non-uniformities of the material along the path of the electron. These variations are taken into account in the identification criteria by defining pseudorapidity-dependent thresholds for the selection variables, in addition to selections dependent on transverse energy. As the tighter *medium* and *tight* criteria make use of both calorimetric and track information, they are more sensitive to such effects. Dependencies are most notable at $|\eta |<0.1$ and in the transition region $1.37<|\eta |<1.52$. In the region $|\eta |>2$, where the requirements on the shower shapes are tightened to preserve the needed rejection in the absence of the transition radiation information, a degradation of the efficiencies is observed.

**Fig. 13:**
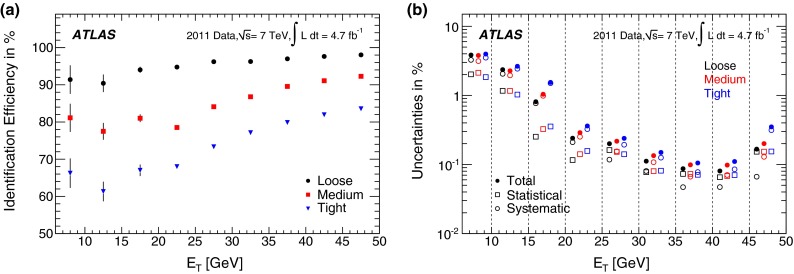
**a** Dependence of the combined identification efficiencies on the transverse energy of the probe for central electrons. *Error bars* correspond to the total uncertainties. **b** Decomposition of the total uncertainty into its statistical and systematic components. The three colours correspond to the three identification criteria (*loose*, *medium*, *tight*). Some points are slightly shifted horizontally within the $E_{\mathrm {T}}$ bin for better visibility. In the $E_{\mathrm {T}}$ region above the Jacobian peak ($E_{\mathrm {T}}>45$ GeV), both the statistical and systematic uncertainties increase with respect to the highest precision region $(E_{\mathrm {T}}\sim 35~\hbox {GeV}),$ as shown in Fig. 12

The dependence of the efficiency on the transverse energy of the electron is made more explicit when integrating over the whole pseudorapidity range of the $Z \rightarrow ee$ sample, as shown in Fig. 13a. In the $E_{\mathrm {T}}$ range from 7 to 50 GeV, the *loose* efficiency varies from about 90 to $98~\%$. The *medium* and *tight* criteria show a more significant dependence on energy due to the tighter requirements applied to provide the desired background rejection. The efficiency increases from about 80 % at 7 GeV to 90 % at 50 GeV for the *medium* criteria and from about 65 % at 7 GeV to 80 % at 50 GeV for the *tight* criteria. The integration over the pseudorapidity range decreases the statistical and uncorrelated systematic uncertainties of the measurement. However, given that almost half of the systematic uncertainty is correlated amongst all $\eta$ bins, the size of this component does not improve after integration. Thus, the total systematic uncertainties on the efficiency measurements as a function of $E_{\mathrm {T}}$ are dominant over much of the lower $E_{\mathrm {T}}$ range and of comparable size to the statistical uncertainties at high $E_{\mathrm {T}}$  as is shown in Fig. 13b.

### Forward-electron identification efficiency

In the forward region of the calorimeters, the electron identification efficiency is measured with a $Z \rightarrow ee$ sample where a well-isolated $E_{\mathrm {T}}>25$ GeV tag electron satisfying the *tight* requirement is identified in the central region of the calorimeter and the probe cluster with $E_{\mathrm {T}}>20$ GeV is found in the region $2.5<|\eta |<4.9$. The candidate events are required to have a low missing transverse momentum, in order to suppress the contributions from $W \rightarrow e\nu$ background.

**Fig. 14:**
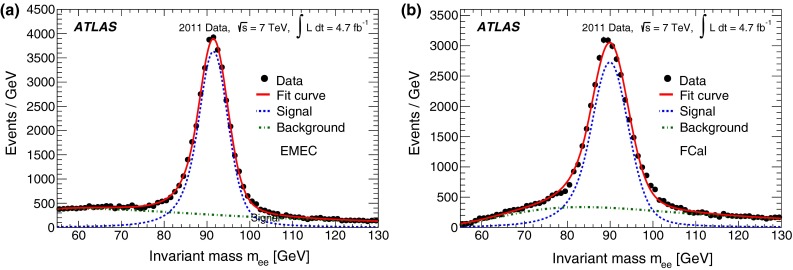
Example fits of invariant mass distributions for probes in the EMEC in **a** and FCal in **b** regions

The invariant mass of the tag–probe system is fitted in each of the pseudorapidity bins defined in Sect. 3.3.2, in the range $55<m_{ee}<130$ GeV to a Crystal Ball function convolved with a non-relativistic Breit–Wigner function with fixed $Z$ width [26] to model the signal, and a Landau function to model the background. The S/B ratio is ${\sim }7$ and ${\sim }5$ in the EMEC and the FCal, respectively. After background subtraction, a total of 192,000 and 76,000 probes remain in the two regions. Variations of the tag requirements are performed, which change the S/B ratio by up to a factor of two. In addition, alternative fit models for signal and background distributions and different fit ranges are used to assess the systematic uncertainties on the electron yields. The total systematic uncertainty is computed by summing in quadrature the effects observed in the individual variations. The largest contributions are related to the choice of background model and signal fit range. Examples of invariant mass fits are shown in Fig. 14.

**Fig. 15:**
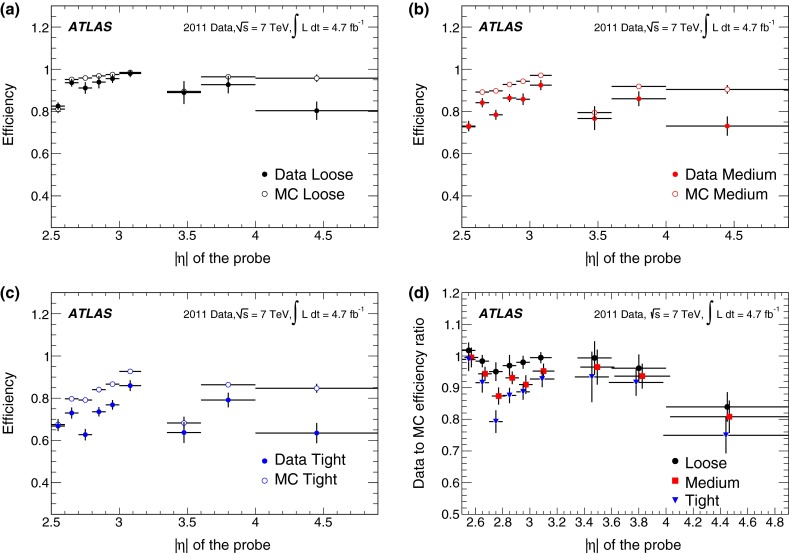
The **a** *loose*, **b** *medium*, and **c** *tight* identification efficiencies as a function of the $|\eta |$ of the probe cluster in the forward region of the calorimeters for data and simulation. In **d**, the data/MC ratio is shown for the three identification criteria, slightly shifted for better visibility. All plots are for probe electrons with $E_{\mathrm {T}}>20$ GeV. In all four figures, *error bars* correspond to the total uncertainties

The electron identification efficiencies measured in data remain stable with increasing pile-up but vary with $E_{\mathrm {T}}$ and $|\eta |$. The simulation models well the measured efficiency shape as a function of pile-up and of $E_{\mathrm {T}}$. However, it does not describe adequately the efficiency measurements as a function of $|\eta |$, as shown in Fig. 15. This discrepancy is due to a mismodelling of the shower shapes in the calorimeter and increases with the tightness of applied identification criteria. Data-to-simulation scale factors are computed in each $|\eta |$ bin to correct for these differences (see Fig. 15d). The resulting total uncertainty is 2–4 and 4–8 % in the EMEC and FCal regions, respectively, and it is dominated by the systematic component.

## Reconstruction efficiency measurement

The EM cluster reconstruction efficiency $\epsilon _\mathrm{cluster}$, for both the central and forward electrons is determined from simulation of $Z \rightarrow ee$ decays. From Sect. 3, the forward EM cluster reconstruction efficiency is better than 99 % for $E_{\mathrm {T}}>20$ GeV and the central EM cluster reconstruction efficiency is 97 and 99 % at 7 and 15 GeV, respectively. It then follows that the central-electron reconstruction efficiency as measured in data reflects the performance of the track reconstruction and the track–cluster matching procedure. Efficiency values are measured for three event samples:

- all reconstructed electron candidates;
- electron candidates satisfying in addition a requirement on the quality of the matching track; this is to match the probe definition of the $J/\psi \rightarrow ee$ selection used in the electron identification efficiency measurement;
- electron candidates with a good track and satisfying in addition the requirement on the hadronic leakage $R_\mathrm{had}$ defined by the *loose* identification criteria; this is to match the probe definition of the $W \rightarrow e\nu$ and $Z \rightarrow ee$ selections.

For this measurement, only one of the channels available to the identification efficiency measurement, a $Z \rightarrow ee$ sample, can be used. The $Z \rightarrow ee$ event selection follows closely that used for the measurement of the identification efficiency as described in Sect. 6.1.1, with the two exceptions noted in Sect. 4 related to the inclusion of photons in the denominator of the efficiency definition and lack of charge requirements on the tag and probe pairs due to the presence of probe clusters without a matched track.

**Fig. 16:**
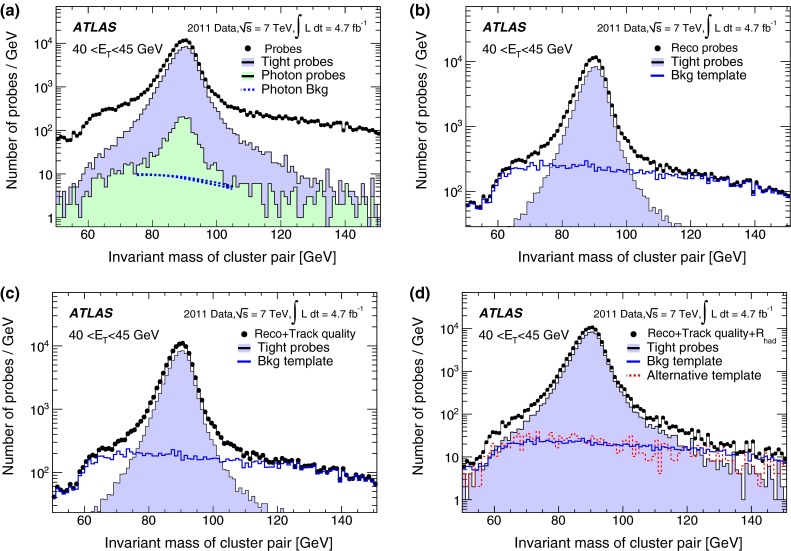
Examples of cluster–pair invariant mass distributions at different levels of the probe selection, in the bin $40\le E_{\mathrm {T}}<45{\mathrm {\ GeV}}$. **a** All reconstructed clusters associated with electrons and photons, used in the denominator of $\epsilon _\mathrm{reco}$; the photon background estimation used to evaluate the corresponding systematic uncertainty is shown. **b** Reconstructed electrons used in the numerator of $\epsilon _\mathrm{reco}$. **c** Reconstructed electrons passing the track quality requirement. **d** Reconstructed electrons passing the track quality and $R_\mathrm{had}$ requirements; two of the different background templates used to estimate the associated systematic uncertainty are shown. In all cases, the *shaded histograms* show the distributions obtained with probes after *tight* identification, to give an indication of the expected signal shape

Figure 16 shows typical examples of the cluster–pair invariant mass distributions used to evaluate $\epsilon _\mathrm{reco}$. A total of 2.2 million probes were used to perform this measurement. The backgrounds entering the numerator and denominator of $\epsilon _\mathrm{reco}$ are evaluated differently due to the inclusion of clusters associated with reconstructed photons in the denominator.

### Background evaluation

The electron background contribution is estimated using a methodology similar to that employed for the identification efficiency measurements discussed in Sect. 6.1.2, based on the electron–positron invariant mass. The background cluster–pair invariant mass template is obtained from data by reversing identification requirements on the probe object and then normalising the distribution to the background-dominated cluster–pair invariant mass distribution in the 110–250 GeV region. When measuring the reconstruction efficiency alone, the probe electrons of the background template are required to fail at least two of the requirements defining the *loose* identification, with the exception of those associated with the track quality, and to satisfy the anti-isolation requirement $E_\mathrm{{T}}^\mathrm{{cone}}(0.4)/E_\mathrm{{T}}> 0.05$ (see Fig. 16b). When measuring the reconstruction efficiency for electrons passing the track quality requirement, the background templates are obtained from objects either passing or failing this extra requirement. The background templates used for the measurement employing the additional requirement on the hadronic leakage $R_\mathrm{had}$ are built in a similar fashion (see Fig. 16c, d).

The background from real photons is estimated using the invariant mass distribution of pairs composed of an electron tag and a probe reconstructed only as a photon, $m_{e\gamma }$. The sideband regions above and below the $Z$-boson resonance mainly contain background events. These regions, corrected for the expected number of genuine electron–positron pairs as estimated from simulation are fit to a third-order polynomial function. The number of background events associated with photons is then obtained by integrating this fit function in the signal region (see Fig. 16a).

### Reconstruction efficiency and systematics

**Fig. 17:**
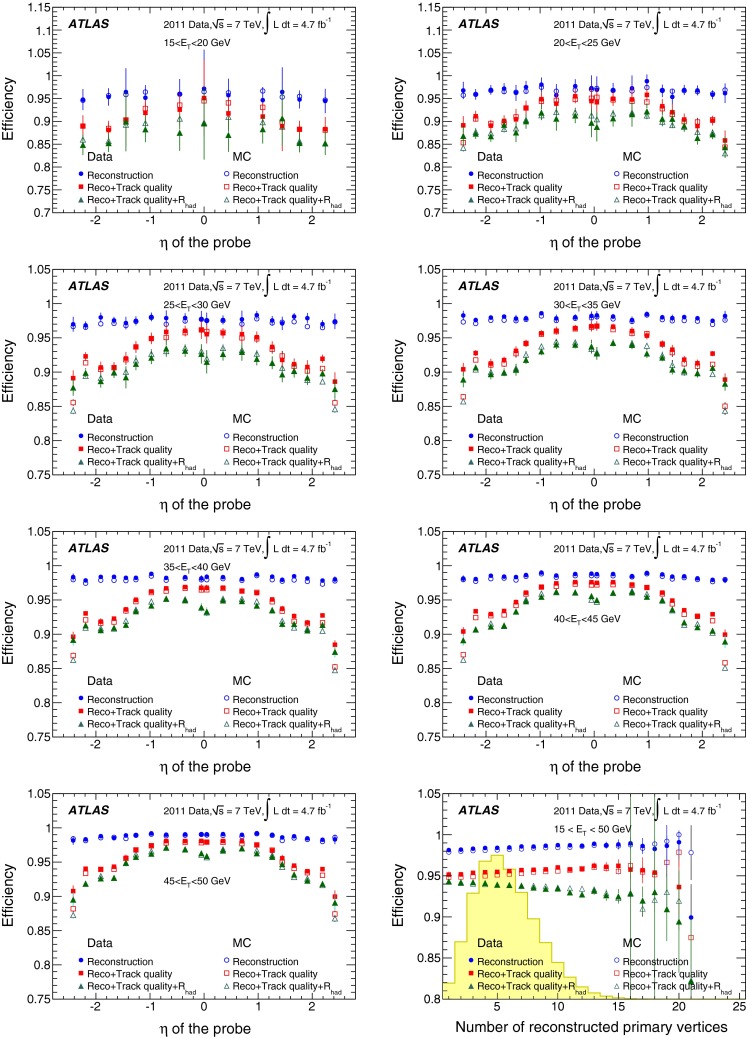
The three types of reconstruction efficiencies, with their total uncertainties, as measured in data and simulation in bins of probe $E_{\mathrm {T}}$ from $15<E_{\mathrm {T}}<20$ GeV to $45<E_{\mathrm {T}}<50$ GeV. The final plot on the bottom right shows the efficiency as a function of the number of reconstructed primary vertices in the event. The *solid yellow histogram* indicates the $N_{\mathrm{PV}}$ distribution in the data. The *error bars* correspond to the total uncertainties

The reconstruction efficiency as a function of pseudorapidity for all three event selections is shown in Fig. 17 in $E_{\mathrm {T}}$ bins ranging from 15 to 50 GeV. In the lowest $E_{\mathrm {T}}$ bin, a coarser $\eta$ binning is used to cope with the smaller data sample, and still ensure that the total uncertainty is equally shared between statistical and systematic sources.

The systematic uncertainties are assessed by varying parameters in the fitting procedure and measuring the global systematic uncertainty as the RMS of the distribution of the results obtained with each configuration. These variations include identification quality of the tag electron, the invariant-mass range used to select the signal events, the template shape used for electrons, and the sideband fit range for the photon background evaluation. For this latter uncertainty, the systematic uncertainty associated with the estimate of the genuine electron–positron events in the sideband region is evaluated by varying this number by ${\pm }30$ % in each $m_{e\gamma }$ bin, assuming this variation is fully correlated between bins. The 30 % variation is conservatively estimated from the largest observed difference between data and simulation for the probability with which electrons are misidentified as photons. The signal contamination in the template and in the normalisation region is taken into account by varying the amount of signal leaking into the cluster–pair invariant mass template, and by estimating the signal contamination in all other regions from simulation. Similarly to what is done for the photon background evaluation, the latter prediction is assigned a conservative 20 % uncertainty.

From Fig. 17, the efficiency to reconstruct an electron or positron having a track of good quality and matching an electromagnetic cluster that fulfils the $R_\mathrm{had}$ requirement varies for high-$E_{\mathrm {T}}$ probes from about 96 % in the barrel region of the calorimeter, to about 90 % in the endcap region for $E_{\mathrm {T}}>30{\mathrm {\ GeV}}$. For $E_{\mathrm {T}}<25{\mathrm {\ GeV}}$, this efficiency drops to about 93 % (85 %) in the barrel (endcap) region. For $E_{\mathrm {T}}>35{\mathrm {\ GeV}}$, the total uncertainty on the measured reconstruction efficiencies is well below 0.5 %.

The reconstruction efficiency may be affected by the ambient activity resulting from pile-up interactions. The final plot in Fig. 17 shows the values of the three reconstruction efficiencies as a function of the number of reconstructed primary vertices $N_{\mathrm{PV}}$ in the event. The $R_{\mathrm{had}}$ requirement introduces the largest sensitivity to pile-up, demonstrated by the few percent efficiency variation as $N_{\mathrm{PV}}$ varies from 1 to 20; this dependence is well modelled by the simulation.

**Fig. 18:**
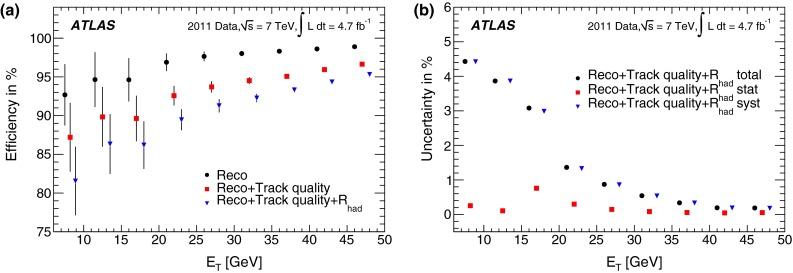
**a** Reconstruction efficiency as a function of $E_{\mathrm {T}}$ for central electrons. The *error bars* correspond to the total uncertainties. **b** Composition of the total uncertainties is shown as a function of $E_{\mathrm {T}}$. For $E_{\mathrm {T}}<15$ GeV no measurement with data was possible and the expected efficiencies from the $Z \rightarrow ee$ MC sample were used directly. In this case, conservative uncertainties of 2 and 5 % were assigned for the barrel and the endcap regions, respectively. Some points are slightly shifted horizontally within the $E_{\mathrm {T}}$ bin for better visibility

The significant background contamination and low statistics of probes at low $E_{\mathrm {T}}$ does not permit a measurement of the reconstruction efficiency for $E_{\mathrm {T}}<15$ GeV from $Z \rightarrow ee$ decays. Furthermore it is not possible to trigger a sufficiently large sample of $J/\psi \rightarrow ee$ or $W \rightarrow e\nu$ events unbiased with respect to the reconstruction efficiency measurement. In the region from 7 to 15 GeV, the prediction from simulation is used instead with fair confidence, based on the observed good MC modelling in the $E_{\mathrm {T}}$ region beyond 15 GeV. For this extrapolation, conservative uncertainties of 2 and 5 % are assigned in the barrel and endcap regions, respectively. Figure 18 shows the three types of reconstruction efficiencies as a function of $E_{\mathrm {T}}$. In the two lowest $E_{\mathrm {T}}$ bins, where no data measurement exists, the expected efficiencies from a $Z \rightarrow ee$ MC sample were used, assigning the systematic uncertainties quoted above. The integration of the measurements over the pseudorapidity range decreases the statistical uncertainty such that the systematic component dominates overs the entire $E_{\mathrm {T}}$ range.

### Combined reconstruction and identification efficiency measurement

**Fig. 19:**
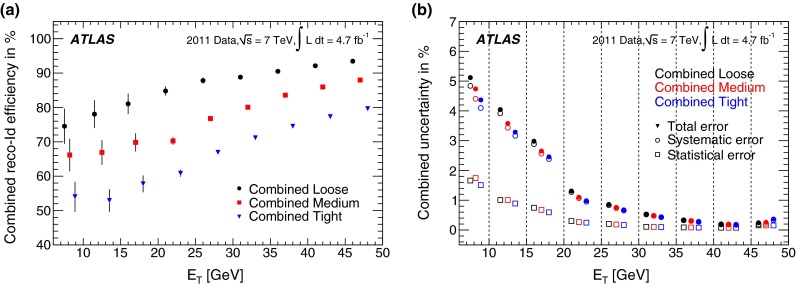
**a** Central-electron combined reconstruction and identification efficiencies as a function of $E_{\mathrm {T}}$, for the reconstruction plus track quality plus hadronic leakage requirements and all three identification criteria. The *error bars* correspond to the total uncertainties. **b** Breakdown of the total uncertainty of the combined measurement into statistical and systematic components as a function of $E_{\mathrm {T}}$. Some points are slightly shifted horizontally within the $E_{\mathrm {T}}$ bin for better visibility

The reconstruction efficiency presented in this section and the identification efficiency in Sect. 6 are combined to provide the electron reconstruction and identification efficiency measurement. This combined efficiency, integrated over the range $|\eta |<2.47$, along with the corresponding uncertainty, is presented as a function of $E_{\mathrm {T}}$ in Fig. 19.

## Charge-identification efficiency

The correct identification of the charge of an electron is important in many analyses, e.g. when exploiting charge correlations of the final-state particles. Electron charge-misidentification may occur when electrons radiate early in the detector, such as near the entrance of the inner tracking detector, and resulting photons subsequently convert and are reconstructed as high $p_{\mathrm {T}}$ tracks. A particle with reconstructed charge opposite to the parent electron may then accidentally be associated with the calorimeter cluster. These effects are expected to follow the distribution of material in the detector, which Fig. 1 shows to be $|\eta |$ dependent.

The probability to correctly identify the charge of the candidate electron is evaluated with a tag-and-probe analysis employing a $Z \rightarrow ee$ sample, considering as probes the ensemble of di-electron pairs without any requirement on the reconstructed sign of the track. The tag is required to satisfy *tight* identification criteria, to be well isolated ($E_\mathrm{{T}}^\mathrm{{cone}}(0.3)/E_\mathrm{{T}}<0.15$) and to have transverse energy greater than 25 GeV. To ensure a well-measured tag charge, the tag is confined to the barrel region of the calorimeter ($|\eta |<1.37$) where the charge reconstruction efficiency is observed to be very high. The probe electron is also required to have $E_{\mathrm {T}}>25$ GeV and be anywhere within the acceptance of the inner detector. No correction is applied for the misidentification of the tight central tag electron. This increases the measured charge-identification probability by about 0.2 %.

The invariant mass of the tag–probe system is used as the discriminating variable to separate signal from background events. The background template is obtained from events that have the tag candidate satisfying the *medium* criteria and the probe candidate failing to satisfy the *loose* criteria. The invariant mass spectrum as measured in data is fit in the region $66<m_{\mathrm {ee}}< 116$ GeV using the sum of the $Z \rightarrow ee$ signal template from simulation and the background template from data. The yield in the signal region is counted in the invariant mass range of 80 to 101 GeV.

**Fig. 20:**
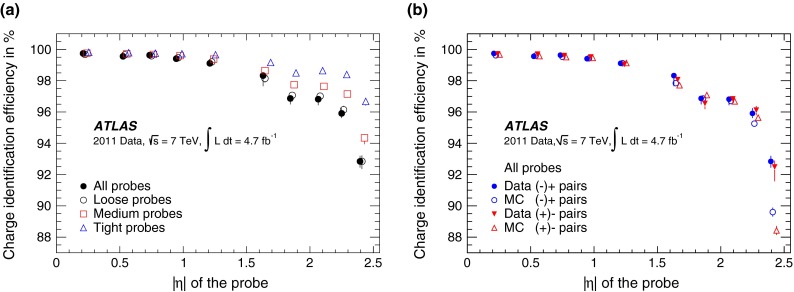
Charge-identification efficiency for electrons with $E_{\mathrm {T}}>25$ GeV in a $Z \rightarrow ee$ sample given as a function of the probe $|\eta |$. **a** Measurement of the data charge-identification efficiencies for reconstructed, *loose*, *medium*, and *tight* probes. **b** Comparison of the efficiencies for all electron and positron probes as measured in data (*closed points*) and in simulation (*open points*). The *sign within the bracket* is the charge of the tag while the sign next to it is that of the probe. The *error bars* correspond to the total uncertainties. Some points are slightly shifted horizontally within the $\eta$ bin for better visibility

The charge-identification efficiency, extracted by comparing this yield to the subset of opposite-sign pairs, is measured for all levels of electron identification: reconstruction, *loose*, *medium*, and *tight*, as shown in Fig. 20a and compared with the equivalent numbers as extracted from simulation in Fig. 20b. The tightness of the tag selection and the definition of the signal region are varied to assess the systematic uncertainty. The charge-identification efficiency is found to be high (${>}99.7$ %) and relatively constant in the barrel region of the calorimeter decreasing to 93 % in the endcap region. In this region, the efficiency increases to 97 % when applying the *tight* selection to the probes. The agreement between data and simulation is good for all $\eta$ values except at the outermost edge of the acceptance where the simulation predicts a higher misidentification probability. This discrepancy may originate from incorrectly modelled material in the simulation. The same figure shows that the measured efficiencies do not depend on the reconstructed sign of the probe track.

## Summary

The ATLAS experiment at the LHC recorded approximately 4.7 fb$^{-1}$ of proton–proton collision data in 2011 at a centre-of-mass energy of $\sqrt{s}=7$ TeV. The tag-and-probe methods developed to measure the components of the electron efficiency with these data are described in detail. In comparison to similar results based on 2010 data [7], the revised analysis methods presented here, in combination with the higher statistics provided by the 2011 data in the $Z \rightarrow ee$, $W \rightarrow e\nu$ and $J/\psi \rightarrow ee$ channels, have enabled precision measurements of electron efficiency in a finely grained two-dimensional grid of probe electron $(E_{\mathrm {T}},\eta ).$

The electron reconstruction efficiency, which is related to the ability to associate a candidate electron track with a corresponding EM cluster, was extracted from a $Z \rightarrow ee$ sample of probe electrons in the central region of the detector ($|\eta |<2.47$) using a fine $\eta$ granularity and seven $E_{\mathrm {T}}$ bins in the range of 15 to 50 GeV. The statistical precision is the dominant source of uncertainty of the two-dimensional measurement, with the total uncertainty varying from a few percent in the lowest $E_{\mathrm {T}}$ bin to ${\sim }0.5$ % at 35 GeV.

The efficiency to identify electrons given the existence of a reconstructed-electron candidate is assessed in the central region of the detector ($|\eta |<2.47$) for three benchmark selection criteria called *loose*, *medium*, and *tight*. A combination of the data to Monte Carlo efficiency ratios measured from $Z \rightarrow ee$, $W \rightarrow e\nu$, and $J/\psi \rightarrow ee$ samples is performed in a fine $(E_{\mathrm {T}},\eta )$ grid, over the probe $E_{\mathrm {T}}$ range from 7 to 50 GeV. This results in a typical accuracy on the efficiency to identify electrons from Z decays of a few per mil at $E_{\mathrm {T}}=35$ GeV and 1–2 % for $E_{\mathrm {T}}<20$ GeV and it is dominated by the statistical uncertainty. As a consequence of improvements in the simulation, the measured efficiencies demonstrate better agreement with expectations compared to the results presented in Ref. [7], varying with $E_{\mathrm {T}}$ and $\eta$ from a few per mil to a few percent. In the forward region ($2.5<|\eta |<4.9$), the efficiency of the entirely calorimeter-based *loose*, *medium*, and *tight* criteria was measured in nine $|\eta |$ bins for probe $E_{\mathrm {T}}>20$ GeV with a total uncertainty of few percent, mostly arising from systematic effects. In this region, a larger discrepancy is observed between measured and expected efficiencies.

The efficiency for a correct charge reconstruction for *tight* electrons with $E_{\mathrm {T}}>25$ GeV is found from a $Z \rightarrow ee$ sample to be ${>}99.7$ % in the barrel region of the detector, decreasing to ${\sim }97$ % in the endcaps, independent of lepton charge.

Overall, the work presented in this paper has enabled precision measurements of two-dimensional efficiencies, improving by approximately an order of magnitude the uncertainties assigned to the results presented in Ref. [7]. These improvements have greatly benefited the analyses performed by the ATLAS collaboration.

## Footnotes

1 Throughout this paper, the term “electron” usually indicates both electrons and positrons.

2 ATLAS uses a right-handed coordinate system with its origin at the nominal interaction point (IP) in the centre of the detector and the $z$-axis along the beam-pipe. The $x$-axis points from the IP to the centre of the LHC ring, and the $y$-axis points upward. Cylindrical coordinates $(r,\phi )$ are used in the transverse plane, $\phi$ being the azimuthal angle around the beam-pipe. The pseudorapidity is defined in terms of the polar angle $\theta$ as $\eta =-\ln \tan (\theta /2)$. Transverse momenta and energies are defined as $p_{\mathrm {T}}=p\sin \theta$ and $E_{\mathrm {T}}=E\sin \theta$, respectively.

3 The cluster moment of degree $n$ for a variable $x$ is defined as:

$$\begin{aligned} \langle x^n\rangle =\left[ \sum _i{E_i\,x_i^n}\right] /\left[ \sum _i{E_i}\right] , \end{aligned}$$

where $i$ is the cell index within the cluster.

4 Results in the high transverse energy region $E_{\mathrm {T}}>50$ GeV are discussed in Ref. [15].

5 In a collider event, the missing transverse momentum is defined as the momentum imbalance in the plane transverse to the beam axis and is obtained from the negative vector sum of the momenta of all particles detected in the event.

6
$m_\mathrm{T}=\sqrt{2E_{\mathrm {T}}E_{\mathrm {T}}^{\mathrm {miss}}(1-\cos \varDelta \phi )}$ where $\varDelta \phi$ is the azimuthal separation between the directions of the electron and missing transverse momentum.

7 The pseudo-proper time is defined as $t_{0}=L_\mathrm{xy}\cdot m^{J/\psi }_\mathrm{PDG}/p_\mathrm{T}^{J/\psi }$, where $L_\mathrm{xy}$ is the displacement of the $J/\psi$ vertex with respect to the primary vertex projected onto the flight direction of the $J/\psi$ in the transverse plane, $m^{J/\psi }_\mathrm{PDG}$ is the nominal $J/\psi$ mass [26] and $p_\mathrm{T}^{J/\psi }$ is the $J/\psi$ reconstructed transverse momentum.

8 Tighter TRT and isolation requirements are applied on the probe samples entering in both the numerator and denominator of the efficiency ratio; both criteria were verified in simulation not to affect the measured identification efficiency.

9
$R_{\phi }$ is the ratio of the energy contained in 3$\times$3 in ($\eta \times \phi$) cells, to the energy in 3$\times$7 cells, computed in the middle layer of the EM calorimeter.

10 The pull gives the deviation from the average value of a measurement in units of standard deviation.
